# ﻿Taxonomic revision of the *Nitocraaffinis* Gurney, 1927 species complex (Harpacticoida, Ameiridae) with descriptions of four new species and re-evaluation of its subspecies

**DOI:** 10.3897/zookeys.1191.115545

**Published:** 2024-02-07

**Authors:** Nuran Özlem Yıldız, Süphan Karaytuğ

**Affiliations:** 1 Institute of Marine Sciences and Technology, Dokuz Eylül University, 35330 İzmir, Turkiye Dokuz Eylül University İzmir Turkiye; 2 Faculty of Science, Department of Biology, Mersin University, 33343 Mersin, Turkiye Mersin University Mersin Turkiye

**Keywords:** Copepoda, marine habitat, meiofauna, microcharacters, new species, taxonomy

## Abstract

Due to the recent increasing importance of microcharacters in copepod taxonomy, it has become evident that many species lacking detailed descriptions actually constitute to a species complex. In this study, *Nitocraaffinis* is redescribed based on lectotype material from Lake Timsah (Egypt) which facilitated a thorough detailed comparison with specimens of *N.affinis* recorded from distantly related localities. The results unequivocally revealed that the specimens of *N.affinis* examined in this study belong to a different species. As a result, four new species, *Nitocrasonmezi***sp. nov.** and *Nitocraserdarsaki***sp. nov.** from the Turkish coast, *Nitocraalperi***sp. nov.** from the Indian Ocean, and *Nitocraloweae***sp. nov.** from Brighton, England are herein described as new to science. On the other hand, all subspecies of *N.affinis*, namely *N.affinisrijekana* Petkovski, 1954, *N.affiniscalifornica* Lang, 1965, *N.affinisstygia*, Por. 1968, and *N.affiniscolombiensis* Fuentes-Reinés & Suárez-Morales, 2014 are elevated to species rank. An updated key the species of the *affinis* group is also provided.

## ﻿Introduction

The family Ameiridae Boeck, 1865 is ranked third in terms of number of taxa in the Harpacticoida, after Miraciidae Dana, 1846 and Canthocamptidae Sars, 1906, comprising 47 genera and up to 300 species (Corgosinho et al. 2020). Boeck (1865) originally established both *Ameira* Boeck, 1865 and *Nitocra* Boeck, 1865 with limited descriptions. The genus *Nitocra* is represented by 81 valid species and subspecies (Karanovic et al. 2015; Huys 2021; Fuentes-Reinés et al. 2022). The taxonomy of the genus *Nitocra*, similar to numerous other genera within the family, has posed challenges due to the lack of detailed species descriptions and taxonomic information (Karanovic and Pesce 2002). Initially designated as “*Nitokra*” by Boeck (1865), the nomenclature was later amended to “*Nitocra*” (Giesbrecht 1881), a spelling upheld until Bowman (1988) highlighted its erroneous usage. This viewpoint was endorsed by Wells (2007), referencing ICZN (1999: § 33.3.1), which advocates for prevailing name usage, thereby asserting the adoption of “*Nitocra*”.

*Nitocraaffinis* was originally described by Gurney (1927) from Ismailia and Port Tawfiq (Egypt), collected during the Cambridge Expedition to the Suez Canal (1924), who described both sexes based on an undisclosed number of specimens. The distributional range of *N.affinis* has expanded with the description of several subspecies from different ecological environments: *N.affinisrijekana* Petkovski, 1954 was described from the North Adriatic, *N.affinisstygia* Por, 1968 was described from the Red Sea, *N.affiniscalifornica* Lang, 1965 was described from California, and *N.affiniscolombiensis* Fuentes-Reinés & Suárez-Morales, 2014 was described from a coastal lagoon in Colombia (Petkovski 1954; Lang 1965; Por 1968; Fuentes-Reinés and Suárez-Morales 2014).

Considering the distributional range of the species, including its subspecies, the wide morphological variations observed among the populations, and the diversity in their ecological habitats, postulations arise that more than one species (i.e., a species complex) may exist under the name of *N.affinis*, and hence needing an urgent revision. Here, *N.affinis* is partially redescribed based on the only incomplete female specimen collected and deposited at the collection of the NHMUK by R. Gurney himself, and proposed here as lectotype (see below), and several populations previously identified as *N.affinis* from a wide range of habitats and geographic localities were re-examined in detail to morphologically delineate the specific range.

## ﻿Material and methods

Specimens were dissected in glycerin and mounted on slides. All drawings were made using an Olympus BX-51 differential interference contrast microscope with the aid of a camera lucida. Figures were prepared with Adobe Photoshop CC using with a Wacom Intuos Pro Graphical tablet. Huys et al. (1996) was followed for the terminology used in the text.

### ﻿Abbreviations used in the text

**A1** antennule

**A2** antenna

**Ae** aesthetasc

**Exp** exopod

**Enp** endopod

**Exp or enp-1, 2, 3** proximal, middle and distal segments of ramus

**P1–P6** legs 1–6

**NHMUK** Natural History Museum United Kingdom

**TCRC** Turkish Copepod Research Collection

**TÜBİTAK** The Scientific and Technological Research Council of Türkiye

## ﻿Results

**Order Harpacticoida Sars, 1903**

### ﻿Family Ameiridae

Taxon classificationAnimaliaHarpacticoidaAmeiridae

Boeck, 1865

1247CBF4-E1FB-55B4-AFD8-1DF0230AC257

#### Updated diagnosis.

Body semi-cylindrical or cylindrical with prosome composed of cephalothorax with completely fused first pedigerous somite, and three free pedigerous somites with smooth hyaline frills. Urosome five-segmented, comprising the fifth pedigerous somite, genital double-somite and three free abdominal somites. Rostrum small, triangular and defined at base or not. Anal operculum apically with row of robust spinules or smooth. Caudal ramus with seven setae. Antennule five to eight-segmented in female, nine or ten-segmented and geniculate in male, first segment not unusually elongate. Antenna with coxa, allobasis or basis, one-segmented endopod, and one or two-segmented exopod. P1–P4 with one to three-segmented exopod and endopod. The inner spine of basis of P1 hook-like in male. P5 with baseoendopod and separate exopod.

### ﻿Genus Nitocra

Taxon classificationAnimaliaHarpacticoidaAmeiridae

Boeck, 1865

F47AA4E6-60EF-5C35-AA29-D63361CC7901

#### Diagnosis.

Body semi-cylindrical. Rostrum small, triangular and defined at base. Anal operculum apically with row of robust spinules. Caudal rami short, and with seven setae. Antennule eight-segmented in female, nine- or ten-segmented and haplocer in male. Antenna with coxa, allobasis, one-segmented endopod, and one-segmented exopod. Partial suture line between basis and first endopodal segment near base of exopod indicates ancestral segmentation. Exopod one-segmented with three setae. Mandible with coxal gnathobase with coarse teeth ventrally, one unipinnate seta dorsally; palp two-segmented, comprising basis and one-segmented endopod. Maxillular endopod represented by minute but distinct segment with two setae. Exopod absent. Maxilla with two endites on the syncoxa. Maxilliped subchelate; syncoxa with subapical seta; endopod represented by strong claw accompanied at base by a minute naked seta. P1–P4 with three-segmented exopod and three-segmented endopod. Inner spine of basis of P1 hook-like in male. P1 exp-2 with one inner seta, P1 exp-3 with four or five setae. P2–P4 exp-1 without inner setae, exp-2 with one inner seta. P2–P4 without sexual dimorphism. P5 with baseoendopod and separate exopod. Male P6 asymmetrical. Sexual dimorphism in the antennule, the inner spine of P1 basis, the inner distal seta of P3 exp-3, P5 and P6 and urosomal segmentation.

#### Type species.

*Nitocratypica* Boeck, 1865 (type species by indication).

#### Valid species and subspecies.

*N.affinis* Gurney, 1927; *N.arctolongus* Shen & Tai, 1973; *N.australis* Soyer, 1975; *N.balli* Rouch, 1972; *N.balnearia* Por, 1964; *N.bdellurae* (Lidell, 1912); *N.bisetosa* Mielke, 1993; *N.blochi* Soyer, 1974; *N.californica* Lang, 1965; *N.cari* Petkovski, 1954; *N.chelifer* Wilson, 1932; *N.colombiensis* Fuentes-Reinés & Suárez-Morales, 2014; *N.delaruei* Soyer, 1974; *N.divaricatacaspica* Behning, 1936; *N.divaricatadivaricata* Chappuis, 1923; *N.dubia* Sars, 1927; *N.elegans* (Scott, 1905); *N.elongata* Marcus, 1968; *N.esbe* Karanovic, Eberhard, Cooper & Guzik, 2014; *N.evergladensis* Bruno & Reid, 2002; *N.fallaciosabaltica* Lang, 1965; *N.fallaciosafallaciosa* Klie, 1937; *N.fragilisfragilis* Sars, 1905; *N.fragilispaulistana* Jakobi, 1956; *N.galapagoensis* Mielke, 1997; *N.gracilimana* Giesbrecht, 1902; *N.hamata* Bodin, 1970; *N.hibernicabulgarica* (Apostolov, 1976); *N.hibernicahibernica* (Brady, 1880); *N.humphreysi* Karanovic & Pesce, 2002; *N.hyperidis* Jakobi, 1956; *N.incerta* (Richard, 1893); *N.intermedia* Pesce, 1983; *N.karanovici* Chullasorn, Kangtia & Klangsin, 2014; *N.kastjanensis* Kornev & Chertoprud, 2008; *N.koreana* Chang, 2007; *N.lacustrisazorica* Kunz, 1983; *N.lacustriscolombianus* Reid, 1988; *N.lacustrislacustris* (Schmankevitsch, 1895); *N.lacustrispacifica* Yeatman, 1983; *N.lacustrisrichardi* Karanovic, Eberhard, Cooper & Guzik, 2014; *N.lacustrissinoi* Marcus & Por, 1961; *N.laingensis* Fiers, 1986; *N.langi* Karanovic, Eberhard, Cooper & Guzik, 2014; *N.malaica* Kiefer, 1929; *N.mediterraneajakubisiaki* Karanovic, Eberhard, Cooper & Guzik, 2014; *N.mediterraneamediterranea* Brian, 1928; *N.medusae* Humes, 1953; *N.minorminor* Willey, 1930; *N.mozambica* Huys, 2021; *N.parafragilis* Roe, 1958; *N.phlegraea* Brehm, 1909; *N.phreatica* Bozic, 1964; *N.pietschmanni* Chappuis, 1934; *N.platypusbakeri* Chappuis, 1930; *N.platypusplatypus* Daday, 1906; *N.pontica* Jakubisiak, 1938; *N.pori* Karanovic, Eberhard, Cooper & Guzik, 2014; *N.psammophila* Noodt, 1952; *N.pseudospinipes* Yeatman, 1983; *N.puebloviejensis* Fuentes-Reinés, Suárez-Morales & Silva-Briano, 2022; *N.pusilla* Sars, 1911; *N.quadriseta* Wells & Rao, 1987; *N.reductafluviatilis* Galhano, 1968; *N.reductareducta* (Schäfer, 1936); *N.reunionensis* Bozic, 1969; *N.rijekana* Petkovski, 1954; *N.sewellihusmanni* Kunz, 1976; *N.sewellisewelli* Gurney, 1927; *N.sphaeromata* Bowman, 1988; *N.spinipesarmata* Lang, 1965; *N.spinipesorientalis* Sewell, 1924; *N.spinipesspinipes* Boeck, 1865; *N.stygia* Por, 1968; *N.taylori* Gómez, Carrasco & Morales-Serna, 2012; *N.typicaadriatica* Petkovski, 1954; *N.typicatypica* Boeck, 1865; *N.uenoi* Miura, 1962; *N.vietnamensis* Tran & Chang, 2012; *N.wolterecki* Brehm, 1909; *N.yeelirrie* Karanovic, Eberhard, Cooper & Guzik, 2014.

### ﻿ Nitocra affinis

Taxon classificationAnimaliaHarpacticoidaAmeiridae

Gurney, 1927

B67CC6D7-FA4C-5C15-98CC-7A7982666686

Figs 1 , 2

#### Unverified records.

Bermuda (Willey 1930), Italia (Chappuis 1938), Sweden (Lang 1935), England (Gurney 1932), Federated States of Micronesia (Vervoort 1964).

#### Type material.

***Lectotype***: Egypt • 1 ♀; Ismailia. Mounted on one slide. Damaged. Abdomen lost. Gurney, R leg.; NHMUK reg. no. 1928.4.2.107.

Gurney (1927) recorded *N.affinis* from both Ismailia and Port Tawfiq and described both sexes based on an undisclosed number of specimens. Since a holotype was not designated by Gurney (1927) all specimens collected from both localities are collectively regarded as the type series. The incomplete female specimen collected and identified by R. Gurney (incorrectly labelled as a co-type) and deposited in the NHMUK under reg. no. 1928.4.2.107 is the only surviving syntype and is here designated as the lectotype of *N.affinis*. The place of origin of the latter is Lake Timsah, Ismailia which becomes the type locality of *N.affinis* according to ICZN Art. 76.2.

#### Redescription

**Figure 1.**
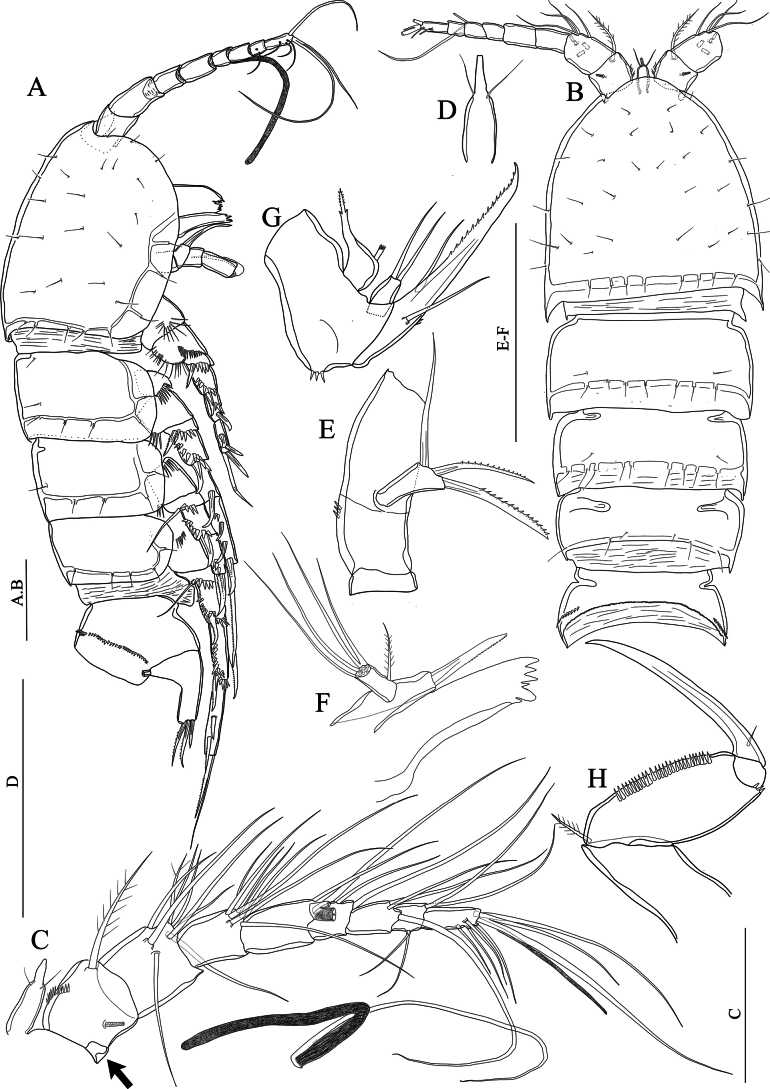
*Nitocraaffinis* female lectotype **A** habitus, lateral **B** habitus, dorsal **C** antennule (arrow pointing pseudosegment) **D** rostrum **E** antennary coxa, allobasis, and exopod **F** mandible **G** maxilla **H** maxilliped. Scale bars: 50 μm.

**(based on the original description and examination of the lectotype).** Prosome slightly tapering proximally with several sensilla as figured (Fig. 1A, B). First urosomite (P5-bearing somite) with lateral spinule row extending to the dorsal edge of the somite. Abdomen missing.

Antennule (Fig. 1C) eight-segmented and joined to the cephalothorax with small triangular pseudosegment (arrowed in Fig. 1C); first segment with spinules at the ventrolateral margin and with a tube pore near the inner dorsal margin, and with row of slender spinules on ventral surface. Long, slender aesthetasc on fourth segment fused basally with adjacent large seta. Two lateral setae on seventh segment and four lateral setae on eighth segment biarticulate at base. Setal formula 1-[1, plumose], 2-[8 +1 plumose], 3-[8], 4-[3 +1 ae], 5-[2], 6-[3], 7-[4], 8-[5 + acrothek)]. Maximum length/maximum width ratio of antennular segments as 1:1.2:1.3:1.8:1.2:1.8:1.3:2.8.

Rostrum (Fig. 1D). Small with two dorsal sensilla near the base of apical rostral projection, which is ~ half of the rostral length, with an opening (pore) distally.

Antenna (Fig. 1E). Coxa small, unornamented. Basis and proximal endopodal segment fused forming allobasis (ancestral segmentation between basis and first endopodal segment visible near base of exopod) with a spinule row near the base of exopod. Exopod one-segmented, with two unipinnate spines and one slender naked seta; endopod lost.

Mandible (Fig. 1F). Gnathobase with coarse teeth ventrally, dorsal unipinnate seta could not be observed due to natural position of structure. Uniramous palp two-segmented comprising basis and one-segmented endopod. Basis armed with a bipinnate spine. Endopod with one plumose lateral seta, and four naked setae (two of them basally fused).

Maxillule not observed. Note: this appendage was impossible to be reliably observed in detail because its position underneath the maxilla. But the structure and setation of the maxillule agrees with the that of *N.loweae* sp. nov. On the other hand, the lectotype material was too fragile and the mouth parts were too small; therefore, the only specimen was not dissected. The maxillule had better be redescribed based on newly collected materials, preferably from newly collected topotype.

Maxilla (Fig. 1G) with two well-developed endites on the syncoxa with a robust row of spinules on outer margin; distal endite with a strong unipinnate seta and two naked setae; proximal endite small, with two naked setae. Allobasis transformed into claw, with one naked seta at base. Endopod a reduced segment with one seta. The maxilla should be redescribed based on newly collected topotypes.

Maxilliped (Fig. 1H) subchelate. Syncoxa unornamented with one subapical plumose seta. Basis ~ 2.4 × as long as maximum width, with row of spinules along inner margin and three small spinules on outer distal corner. Endopod represented by strong claw accompanied at base by a minute naked seta.

**Figure 2.**
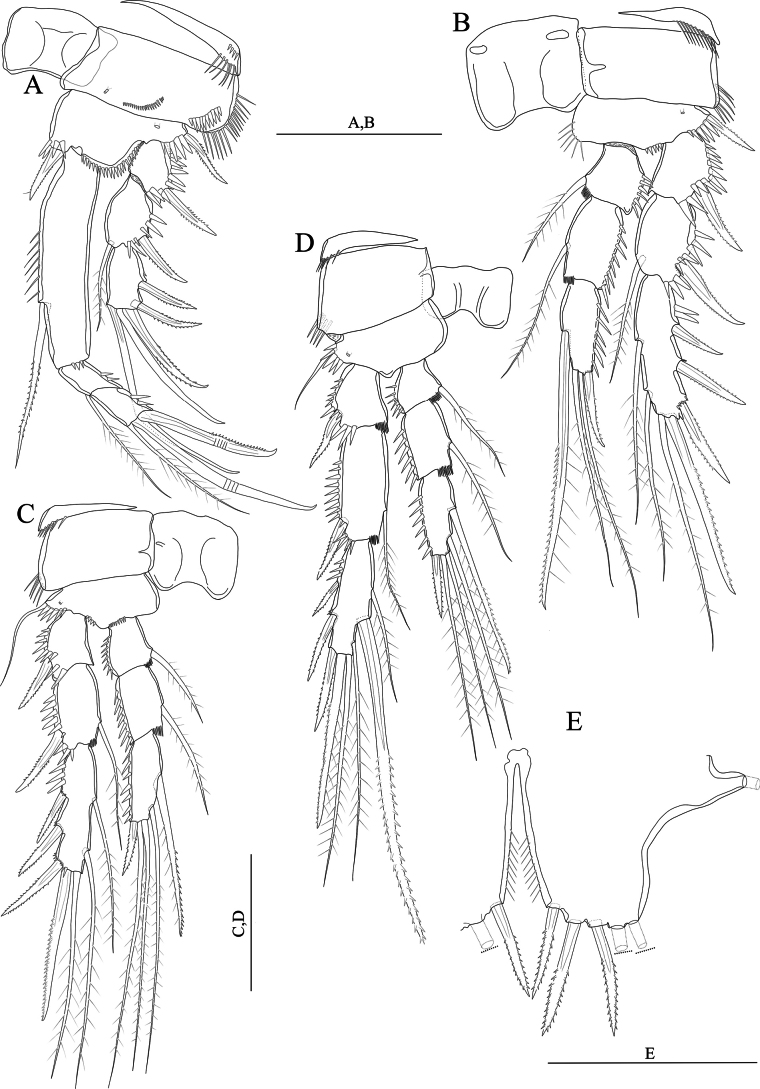
*Nitocraaffinis* female lectotype **A**P1, anterior **B**P2, anterior **C**P3, anterior **D**P4, anterior **E**P5, anterior. Scale bars: 50 μm.

Swimming legs (Fig. 2A–D); P1–P4 with three-segmented exopods and endopods (Fig. 2A–D). Intercoxal sclerite rectangular and smooth. Praecoxa wide and with a row of spinules on outer margin (P1–P4).

P1 (Fig. 2A). Coxa rectangular, outer distal margin ornamented with fine setules; anterior surface with a row of spinules. Basis with spinule row at the base of strong pinnate inner spine; with spinule row along distal margin. Inner margin of basis with robust spinules. Exp-1,2 with outer pinnate spine. Exp-2 with an inner plumose seta. Exp-3 with two geniculate apical setae and three outer pinnate spines. Enp-1 longer than exopod, 5 × as long as maximum width and ornamented with row of fine spinules on the middle of the segment along inner margin, with three spinules on distal margin and with a bipinnate spine originating from the distal half of the segment; enp-2 with a plumose seta on inner corner and with spinules on outer margin; enp-3 with one plumose seta and two geniculate setae distally and with few spinules on outer margin.

P2–P4 (Fig. 2B–D). Coxa rectangular, outer distal margin naked. Inner margin of basis naked (P3, P4) or ornamented with fine setules (P2). Exp-1 without inner seta; (P2–P4) and inner distal margin of exp-1,2 with small spinules (P3, P4). Exp-1,2 with robust spinules and pinnate spine (P2–P4) on outer margin; exp-2 with an inner plumose seta (P2–P4); P2 and P3 exp-3 with seven elements; two inner plumose setae, two apical setae (the outermost being spiniform and unipinnate, the innermost slender and plumose) and three pinnate outer spines. P4 exp-3 with eight elements; two slender inner plumose setae, one well-developed inner pinnate seta, two apical setae, (the outermost being spiniform and unipinnate, the innermost slender and plumose) and three pinnate outer spines. Enp-1, 2 ornamented with robust spinules on outer margin, with small spinules on inner distal margin, and with a plumose inner seta (P2–P4); P2enp-3 with four elements; one proximal inner unipinnate seta, two distal plumose setae and one distal outer spine; P3 and P4enp-3 with five elements; one proximal inner unipinnate seta, one inner subdistal seta, two distal plumose setae and one distal outer spine.

P5 damaged, exopod lost (Fig. 2E). Distal half of inner margin of baseoendopod with setules; endopodal lobe with five setae (two broken off, but these two missing setae depicted in the original description (see Gurney 1927: fig. 154 D, E) Armature formula of the swimming legs as follows:

**Table:** 

P1	P2	P3	P4
Exp/ Enp	Exp/ Enp	Exp/ Enp	Exp/ Enp
0.1.023 / 1.1.111	0.1.223 / 1.1.121	0.1.223 / 1.1.221	0.1.323/1.1.221

### ﻿ Nitocra loweae sp. nov.

Taxon classificationAnimaliaHarpacticoidaAmeiridae

6B8AD5E2-68ED-5B05-AC6F-D070B045776D

https://zoobank.org/4DA4374E-752C-4EFC-8891-C0B25C11256C

**Figure 3.**
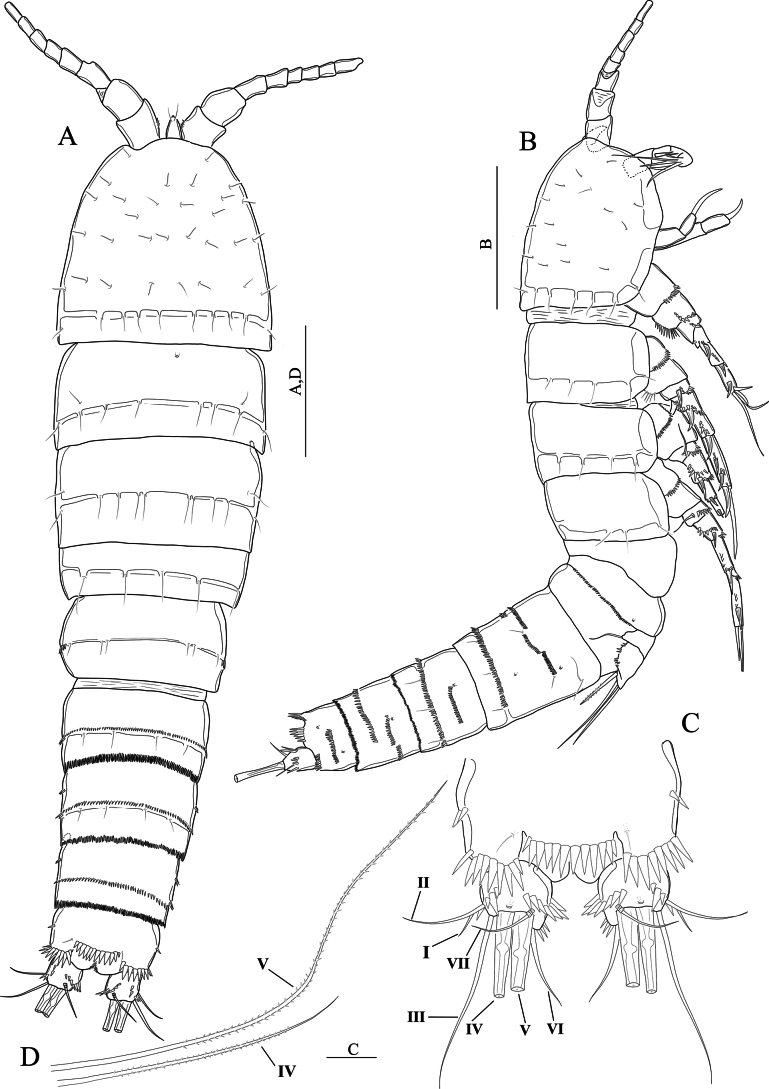
*Nitocraloweae* sp. nov. female holotype **A** habitus, dorsal **B** habitus, lateral **C** anal somite, dorsal **D** furcal setae IV and V. Scale bars: 100 μm (**A, D**); 50 μm (**B**); 12 μm (**C**).

**Figure 4.**
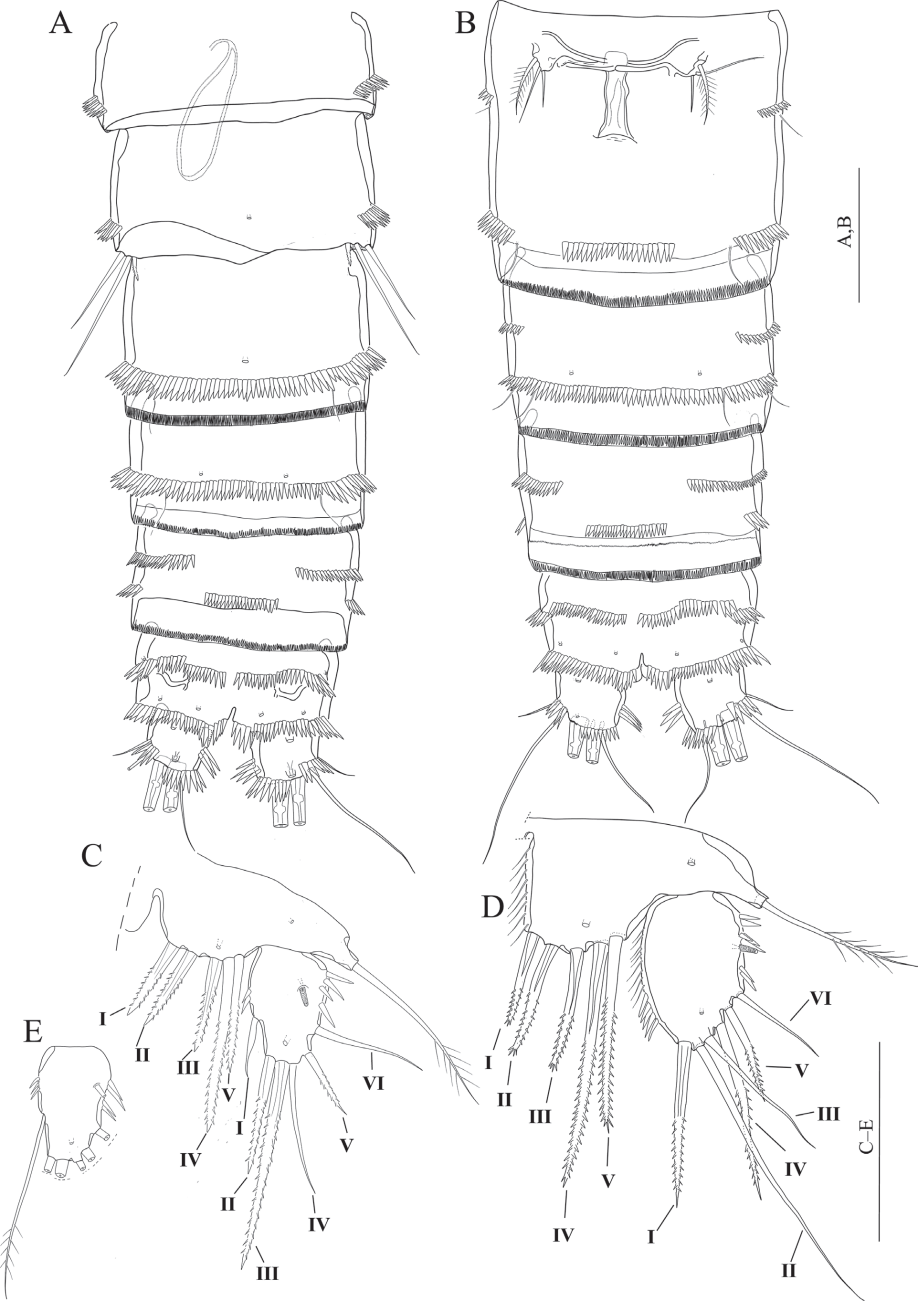
*Nitocraloweae* sp. nov. paratype male (**A, C**), female (**B, D**) **A** urosome, ventral **B** urosome, ventral **C**P5**D**P5, anterior **E** the abnormal inner seta of P5 exopod. Scale bars: 50 μm.

**Figure 5.**
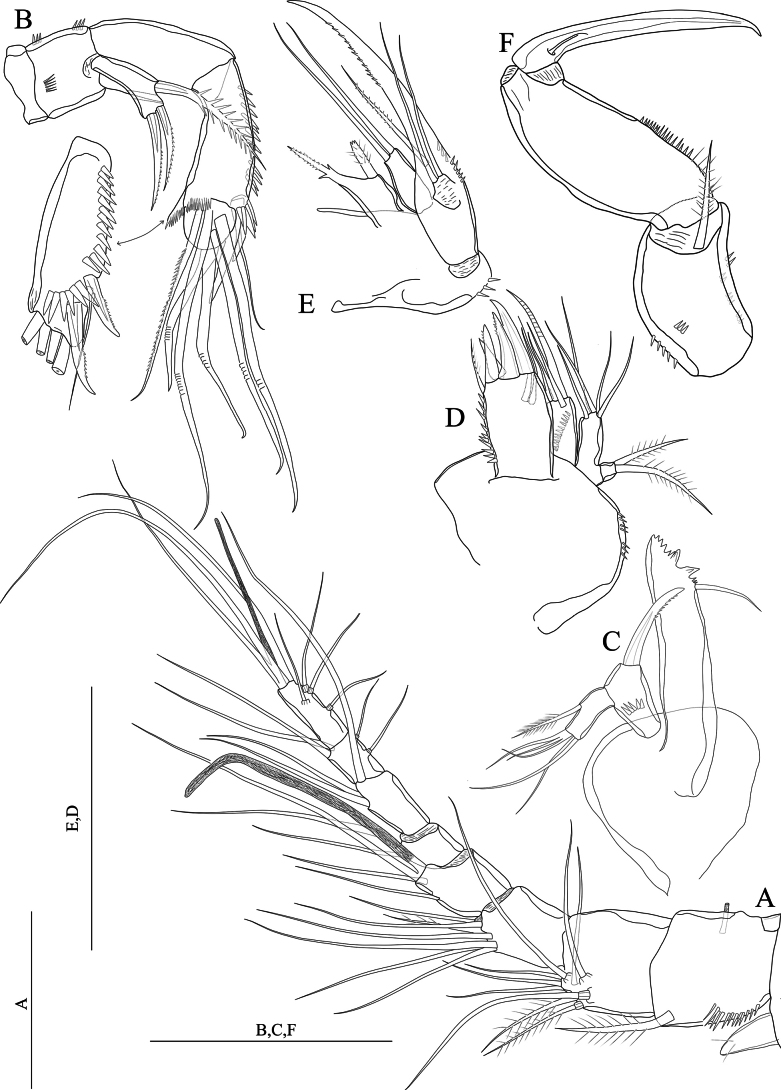
*Nitocraloweae* sp. nov. female holotype **A** antennule **B** antenna, with insert showing free endopodal lobe from another view **C** mandible **D** maxillule **E** maxilla **F** maxilliped. Scale bars: 50 μm.

**Figure 6.**
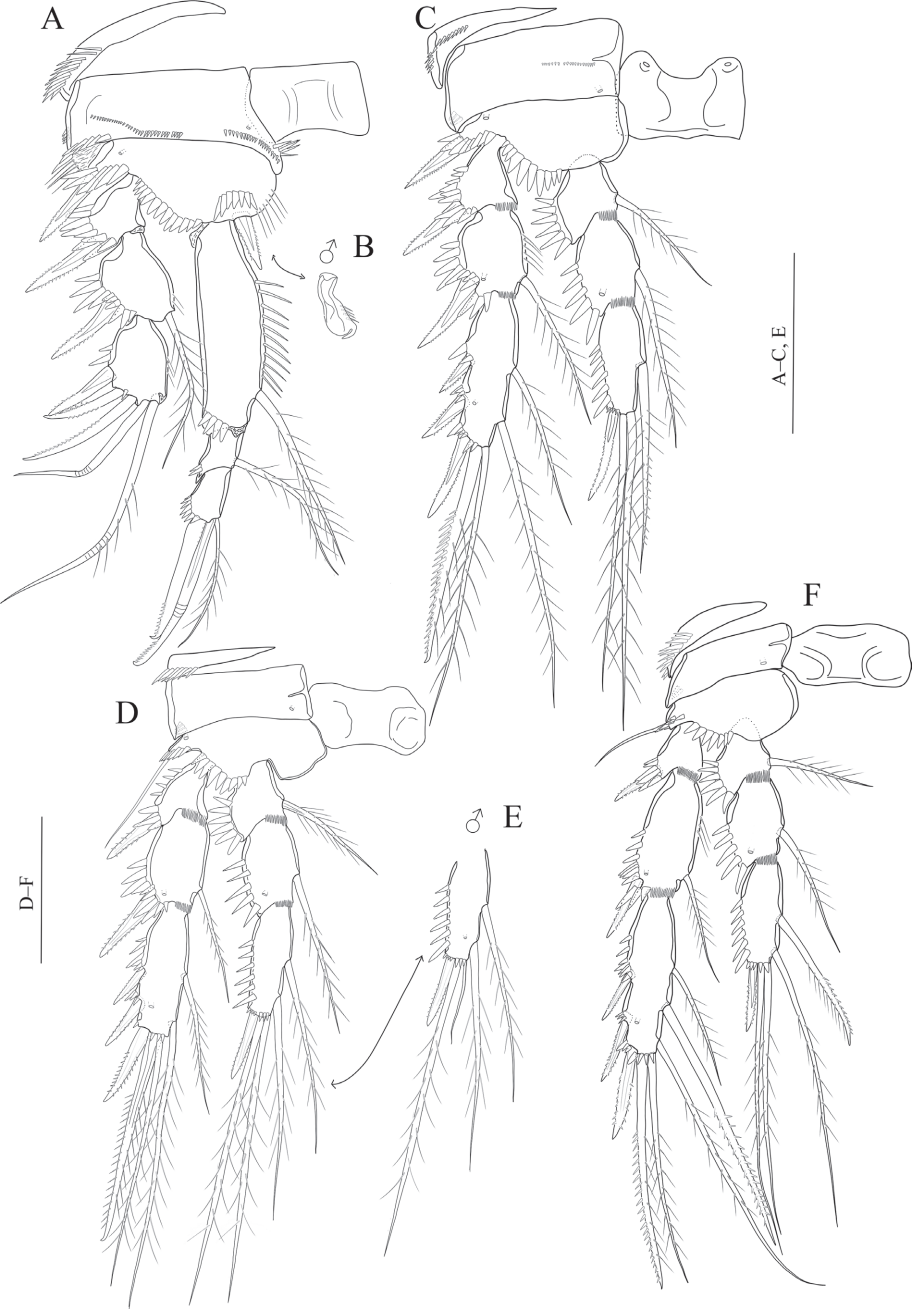
*Nitocraloweae* sp. nov. female holotype (**A, C, D, F**), male paratype (**B, E**) **A**P1**B** inner spine of P1 basis, anterior **C**P2, anterior **D**P3, anterior **E**P3 distal endopod segment, anterior **F**P4, anterior. Scale bars: 50 μm.

**Figure 7.**
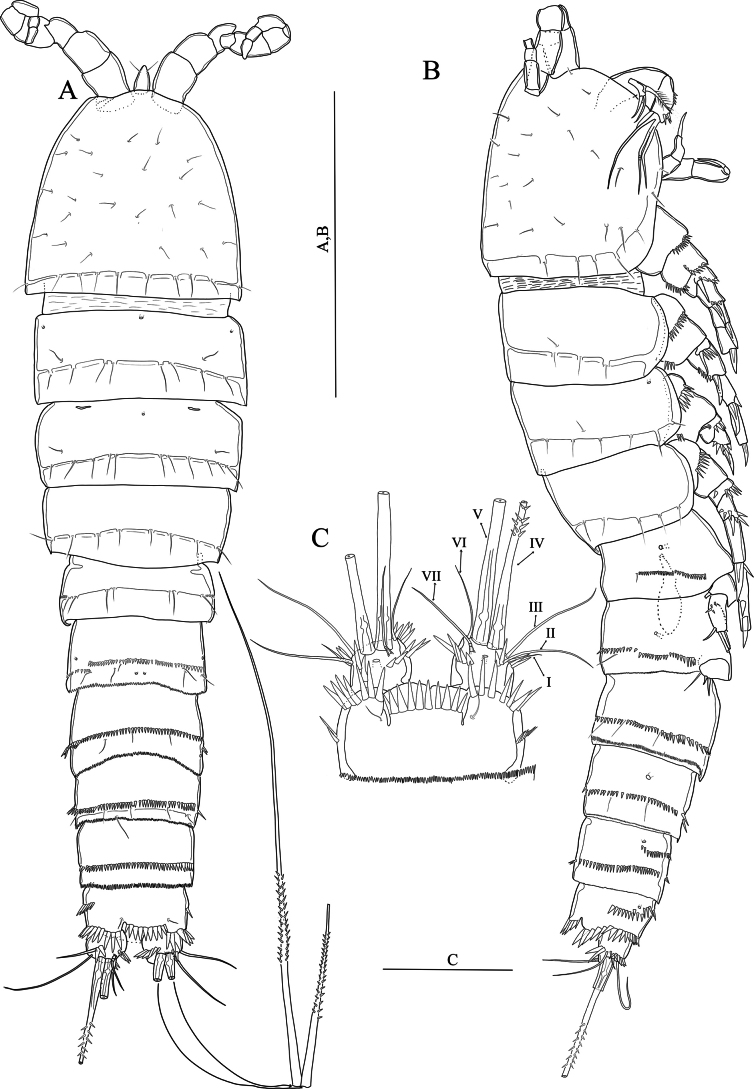
*Nitocraloweae* sp. nov. male paratype **A** habitus, dorsal **B** habitus, lateral **C** anal somite and caudal rami, dorsal. Scale bars: 250 μm (**A, B**); 50 μm(**C**).

**Figure 8.**
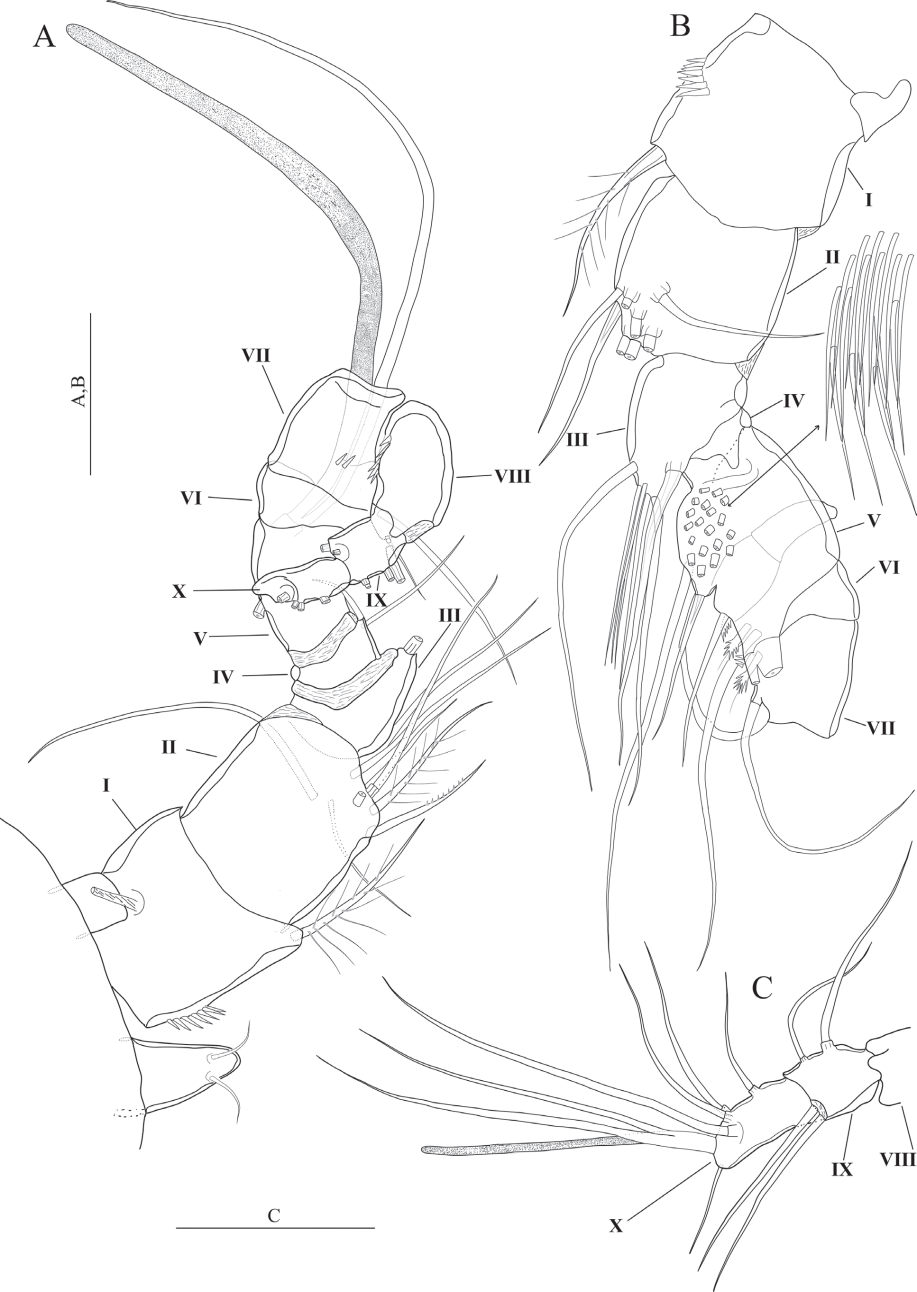
*Nitocraloweae* sp. nov. male paratype **A** antennule dorsal **B** antennule 1–7 segments, ventral **C** antennule 8–10 segments, ventral. Scale bars: 25 μm.

Figs 3 , 4 , 5 , 6 , 7 , 8

#### Type material.

***Holotype***: England • 1 ♀ (dissected on 7 slides) (NHMUK reg. no. 2023.0000); paratype: 1 ♂ (ethanol-preserved) (NHMUK reg. no. 2023.0000); additional paratypes; 2 ♀♀ (ethanol-preserved) (NHMUK reg. no. 2023.0000-0000). Brighton, 50°48.46'N, 00°04.85'W; washings of *Polysiphoniafucoides* algae collected at 1.5 m depth. Leg. David Ventham, 13.10.1993 (material originally registered as *N.affinis* under NHMUK reg. no. 2015.1108) (see Ventham 2011).

#### Description

**(adult female holotype).** Body semi-cylindrical (Fig. 3A, B), total body length measured from the tip of the rostrum to posterior end of the caudal rami 560–571 μm (average = 564.6, *n* = 3; holotype length = 571 μm). Sensilla and pore ornamentation as figured (Fig. 3A, B). Prosome composed of cephalothorax with completely fused first pedigerous somite, and three free pedigerous somites with smooth hyaline frills. Urosome five-segmented, comprising fifth pedigerous somite, genital double-somite and three free abdominal somites. Fifth pedigerous somite wider than other urosomites, with six sensilla and a lateral spinule row slightly extending dorsally. Genital double-somite (Figs 3A, B, 4B) squarish, internal suture line (remnant of segmental fusion) strongly sclerotised, visible dorsolaterally at midlength of somite, ornamented with spinules as figured. Second and third abdominal somites with sensilla and spinules as figured (Figs 3A, B, 4B). Genital field positioned near anterior margin of genital double-somite (Fig. 4B); paired gonopores opening via common midventral slit covered by genital operculum derived from fused vestigial sixth legs. P6 with one plumose seta and one naked seta (Fig. 4B). Copulatory pore large (Fig. 4B), leading via chitinised copulatory duct with supporting chitinised rod. Anal somite (Figs 3A, C; 4B) with a lateral row of spinules medially; ventrally with medial row of spinules (Fig. 4B); anal operculum apically with row of twelve robust spinules (Fig. 3C). Caudal rami (Fig. 3C) with robust spinules near inner margin running dorso-ventrally, and a middorsal pore; with a posterior row of strong spinules ventrally; with seven setae (Fig. 3C): seta I minute; seta II slightly displaced dorsally; setae IV and V well-developed and pinnate (Fig, 3D); seta VI located near inner distal margin and naked; seta VII proximally tri-articulate.

Antennule (Fig. 5A) eight-segmented. Setal formula 1-[1, plumose], 2-[7 +2 plumose], 3-[7 +1 plumose], 4-[3 +1 ae], 5-[2], 6-[3], 7-[4], 8-[5 +acrothek)]. Maximum length/maximum width ratio of antennular segments 1:1:1.4:1.2:1.2:2:1.5:2.8.

Rostrum (Fig. 5A) small, triangular, without clear demarcation between the distal and the proximal part of rostrum (cf. Fig. 1C of *N.affinis*) with tube pore distally and with two dorsal sensilla (Fig. 5A).

Antenna (Fig. 5B) comprising coxa, allobasis, one-segmented endopod and one-segmented exopod.. Coxa very short and unornamented. Allobasis cylindrical, ~ 2.7 × as long as maximum width, ornamented proximally with three spinule rows. Free endopodal segment with proximal part narrower than distal part, ~ 2.5 × as long as its maximum width, ornamented with surface frill subdistally, and with longitudinal row of spinules along inner margin, with another spinule row near the base of two lateral unipinnate spines flanking thin naked seta; apical armature consisting of five geniculate setae, one of them fused basally to additional unipinnate non-geniculate seta. Exopod with narrow proximal half and somewhat wider distal part, ~ 2.5 × as long as its maximum width, unornamented, armed with two curved, strong unipinnate apical setae and one spinulose subdistal seta, the latter longest.

Mandible (Fig. 5C). Coxal gnathobase with coarse teeth ventrally and with one unipinnate seta dorsally. Palp uniramous, two-segmented, comprising basis and one-segmented endopod. Basis with lateral spinule row midway and one curved robust unipinnate apical spine. Endopod with one short plumose lateral seta, five naked apical setae (three of them basally fused).

Maxillule (Fig. 5D). Praecoxa large with few spinules. Praecoxal arthrite rectangular; with two setae on anterior surface, lateral spinule row and distal armature consisting of four spines (two of which with apical combs) and one unipinnate seta. Coxal endite shorter than praecoxal arthrite, with long distally curved spine and three slender naked setae. Basis rectangular, with five slender naked setae on distal margin. Endopod represented by minute but distinct segment, unornamented and armed with two plumose apical setae. Exopod absent.

Maxilla (Fig. 5E). Syncoxa with spinule row and two well-developed (coxal) endites; proximal endite somewhat bulbous, expanded distally and armed with two plumose setae; distal endite cylindrical with two naked apical setae of equal in length. Allobasis transformed into claw-like pinnate endite; with a pinnate seta at base and with few spinules along convex margin near the base of endopod. Endopod represented by two slender naked setae equal in length.

Maxilliped (Fig. 5F) subchelate. Syncoxa with one plumose subapical seta and with spinule rows on posterior surface. Basis ~ 2.6 × as long as maximum width with row of spinules along inner margin and with row of spinules on outer distal corner. Endopod represented by strong claw accompanied at base by minute naked seta.

P1–P4 (Fig. 6A, C, D, F) exopod and endopod three-segmented. Intercoxal sclerite squarish and unornamented. Praecoxa triangular, outer margin with row of spinules. Exp-1 without inner seta. P1, P2 and P4 exp-2 with few, P3 exp-2 without few spinules along inner margin.

P1 (Fig. 6A). Coxa with anterior row of spinules as figured and few spinules along inner margin. Basis with fine setules along inner margin, and with spinule row near the base of short pinnate inner spine located at the base of endopod. Outer basal bipinnate spine located close to the exopod, overlaid by a row of strong spinules. Exp-1 smallest segment, carrying row of strong spinules on outer margin and subapically one bipinnate outer spine. Exp-2 also with an outer row of strong spinules, a bipinnate outer spine, and a long plumose seta on inner margin. Exp-3 with fine setules near inner distal margin, with one geniculate semi-plumose apical seta, one geniculate naked distal seta and three pinnate outer spines. Enp-1 longer than exopod, ~ 3 × as long as maximum width, and ~ 2 × as long as enp-1, 2 combined; inner margin with long longitudinal spinules; distal margin with four robust spinules, and a long plumose seta on inner subdistal margin; enp-2, 3 squarish and equal in length; enp-2 with a long, plumose seta on inner margin, and with two spinules on outer distal margin; enp-3 with one plumose inner seta, one apical unipinnate distal geniculate seta, and one apical unipinnate outer seta, outer margin with a row of spinules.

P2–P4 (Fig. 6C, D, F). Coxa with row of posterior spinules near outer margin and with an anterior pore near inner distal corner. Basis triangular, ornamented with spinule row along distal margin between the base of exopod and endopod, with anterior spinules on outer corner near the base of outer seta/spine. Exp-1, 2 with anterior spinules near the base of outer spine extending to the outer margin of the segment. Exp-1, 2 and enp-1, 2 with hyaline frills along inner distal margin. All endopodal segments covered with robust spinules along outer margin. Exp-2, 3 and enp-2 with a pore on anterior surface. P2–P4 exp-2 with one plumose seta; P2, P3 exp-3 with seven, P4 exp-3 with eight elements.

P5 (Fig. 4D). Baseoendopod with five spinulose setae (slightly fringed at tip) along distal margin and with setules along inner margin, with two anterior pores (one near the base of outer basal seta and the other one near the distal margin; outer basal seta plumose. Exopod with one anterior pore distally, with setules along inner margin and with robust spinules along distal margin, with six setae (setae I–VI, numbered from inner to outer margin respectively), setae I, IV, and V pinnate; setae II, III, and VI naked; seta II is the longest; outer margin of exopodal lobe with one tube pore, and with double spinules group.

Armature formula of the swimming legs:

**Table:** 

P1	P2	P3	P4
Exp/ Enp	Exp/ Enp	Exp/ Enp	Exp/ Enp
0.1.023 / 1.1.111	0.1.223 / 1.1.121	0.1.223 / 1.1.221	0.1.323/1.1.221

**Male.** Body sensilla and surface pores as figured (Fig. 7A, B). Anal operculum with sensilla and spinules as figured (Fig. 7C). Sexual dimorphism in antennule (Fig. 8A–C), inner spine of P1 basis (Fig. 6B), inner distal seta of P3 exp-3 (Fig. 6E), P5 (Fig. 4C, D), and P6 (Fig. 4A). The innermost seta of P5 is abnormal in paratype (Fig. 4E).

Antennule (Fig. 8A–C), ten-segmented, setal formula; 1- [1, plumose], 2- [1 plumose+ 1 unipinnate+ 8], 3-[7], 4-[2], 5-[19 setiform elements+ 4 multipinnate spine+3+1+ae], 6- [2], 7- [3], 8-[2], 9-[5], 10- [5 +acrothek)].

Inner spine of basis of P1 hook-like (Fig. 6B).

Inner distal seta of P3 exp-3 (Fig. 6E) slender and shorter than in female.

P5 (Fig. 4C) baseoendopod armed with five spinulose setae (four of them equal in length, the second inner seta longest and 1.5 × as long as the other setae) and with two pores on anterior surface; exopod with six setae (outer margin with one naked seta (seta VI) and one spinulose seta (seta V), apical margin with one naked (seta IV) and two spinulose setae (setae II and III), inner margin with one long plumose seta (seta I) (abnormal seta of one leg arrowed in Fig. 4C; the same seta of the other leg normal), with four strong spinules along outer proximal margin and with two or three spinules along inner proximal margin.

P6 (Fig. 4A) asymmetrical, only one leg functional; each leg with two naked outer setae and short inner plumose robust seta.

#### Etymology.

The specific name is given in honour of Dr Miranda Lowe for her contribution to copepod taxonomy as a curator of the Crustacea collection of The Natural History Museum of London. It is a noun in the genitive case.

### ﻿ Nitocra sonmezi sp. nov.

Taxon classificationAnimaliaHarpacticoidaAmeiridae

E8B132A8-02D6-5911-864E-9F99F62B18E4

https://zoobank.org/8D69B94B-57EF-44DC-B356-FF245B506028

Figs 9 , 10 , 11

#### Type material.

***Holotype***: Türkiye • 1 ♀ (dissected on 9 slides) (reg. no. TCRC-2007/10). Hatay Province Arsuz (Mağaracık); 36°14.008'N, 35°50.220'E; 24/11/2007 collected from interstitial habitat; leg. Drs Serdar Sönmez, Alp Alper, Serdar Sak, Süphan Karaytuğ (this specimen was previously deposited in the collection of Biology Department of Mersin University and was labelled as *N.affinis* as a result of the faunistic project from Mediterranean Sea, under the project number TÜBİTAK TBAG-106T590).

#### Description

**Figure 9.**
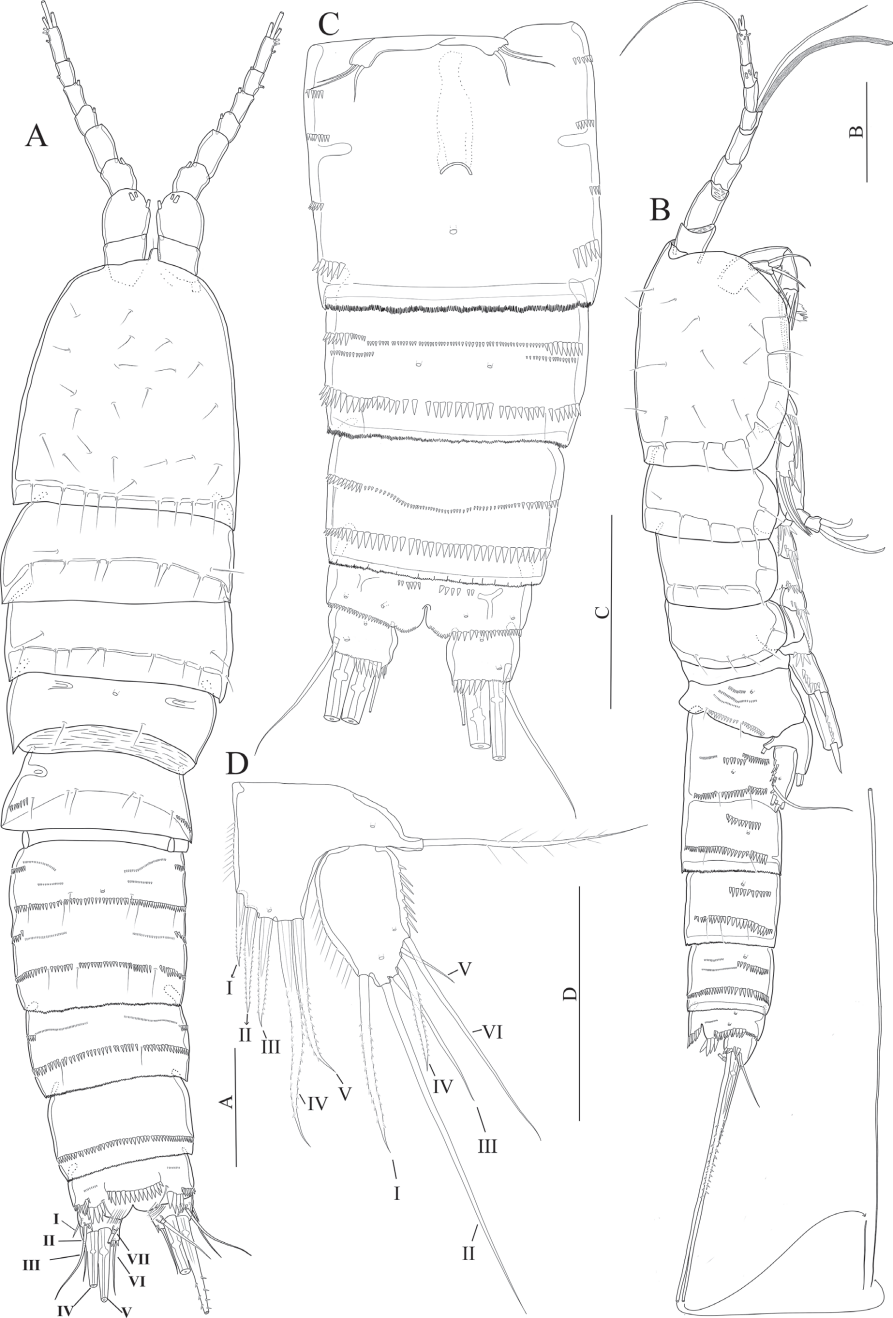
*Nitocrasonmezi* sp. nov. female holotype **A** habitus, dorsal **B** habitus, lateral **C** urosome, ventral (P5-bearing somite omitted) **D**P5, anterior. Scale bars: 50 μm.

**(adult female holotype).** Body (Fig. 9A) semicylindrical; total body length measured from tip of the rostrum to posterior end of the caudal rami 400 μm (*n* = 1). Surface sensilla and pores as figured (Fig. 9A, B). Urosomites with finely serrated hyaline frills, and with complex spinule rows as figured (Fig. 9A–C). Genital double-somite (Fig. 9A, C) viewed as squarish in dorsal and ventral view, rectangular in lateral view (Fig. 9B), with lateral suture line; with two continuous spinule rows dorsally extending laterally as figured. Anal somite (Fig. 9A, C) with two pores located ventrolaterally and medially, anal operculum with fifteen robust spinules. Caudal rami (Fig. 9A, C) short and squarish; with fine setules near the base of seta VII, with row of spinules dorsally near the base of seta II; few spinules present around inner distal margin; with seven setae: seta I minute; seta II slightly displaced dorsally; setae IV and V well-developed and pinnate; seta VI located near inner distal margin and naked; seta VII tri-articulate at base.

Antennule eight-segmented, setal formula 1-[1, plumose], 2-[8 +1 plumose], 3-[8], 4-[3 +1 ae], 5-[2], 6-[3], 7-[4], 8-[5 +acrothek)]. Maximum length/maximum width ratio of antennular segments 1:1.1:1.4:1.8:1.2:2:1.4:2.8.

**Figure 10.**
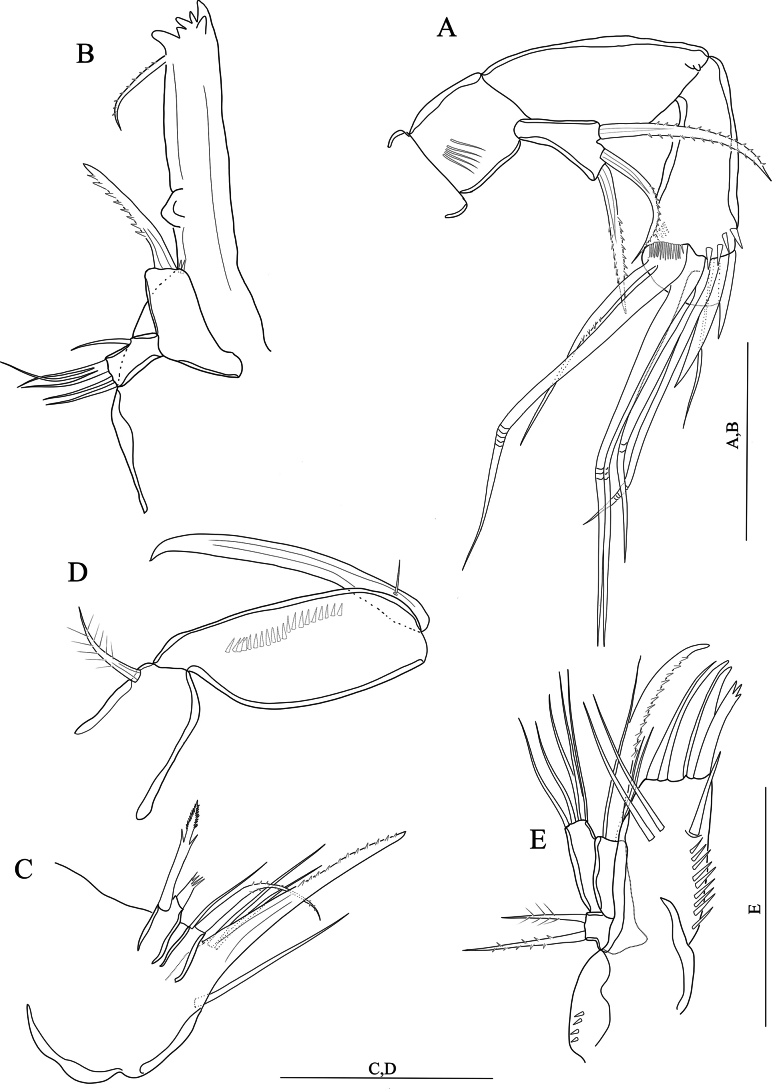
*Nitocrasonmezi* sp. nov. female holotype **A** antenna **B** mandible **C** maxilla **D** maxilliped **E** maxillule. Scale bars: 25 μm.

Antenna (Fig. 10A) comprising coxa, allobasis, one-segmented endopod and one-segmented exopod same as in *N.loweae* sp. nov. except for allobasis without spinule rows proximally. Endopod without longitudinal row of spinules along inner margin. Exopod with a weakly pinnate subdistal seta.

Mandible (Fig. 10B) coxal gnathobase with coarse teeth ventrally, one unipinnate seta dorsally, palp uniramous; two-segmented, comprising basis and one-segmented endopod same as in *N.loweae* sp. nov. except for endopod lateral seta is naked, basis without lateral spinule row.

Maxilla (Fig. 10C) with syncoxa and two well-developed endites same as in *N.loweae* sp. nov. except for syncoxa without spinule row; distal endite of syncoxa cylindrical with one naked, and one stronger and longer semi-pinnate apical setae, allobasis without spinules along convex margin near the base of endopod.

Maxilliped (Fig. 10D) subchelate same as in *N.loweae* sp. nov. except for syncoxa unornamented; basis without spinules on outer distal corner.

Maxillule (Fig. 10E) praecoxa, coxal endite, basis same as in *N.loweae* sp. nov. except for endopod with one plumose and one bipinnate seta, curved seta of coxal endite long and unipinnate.

**Figure 11.**
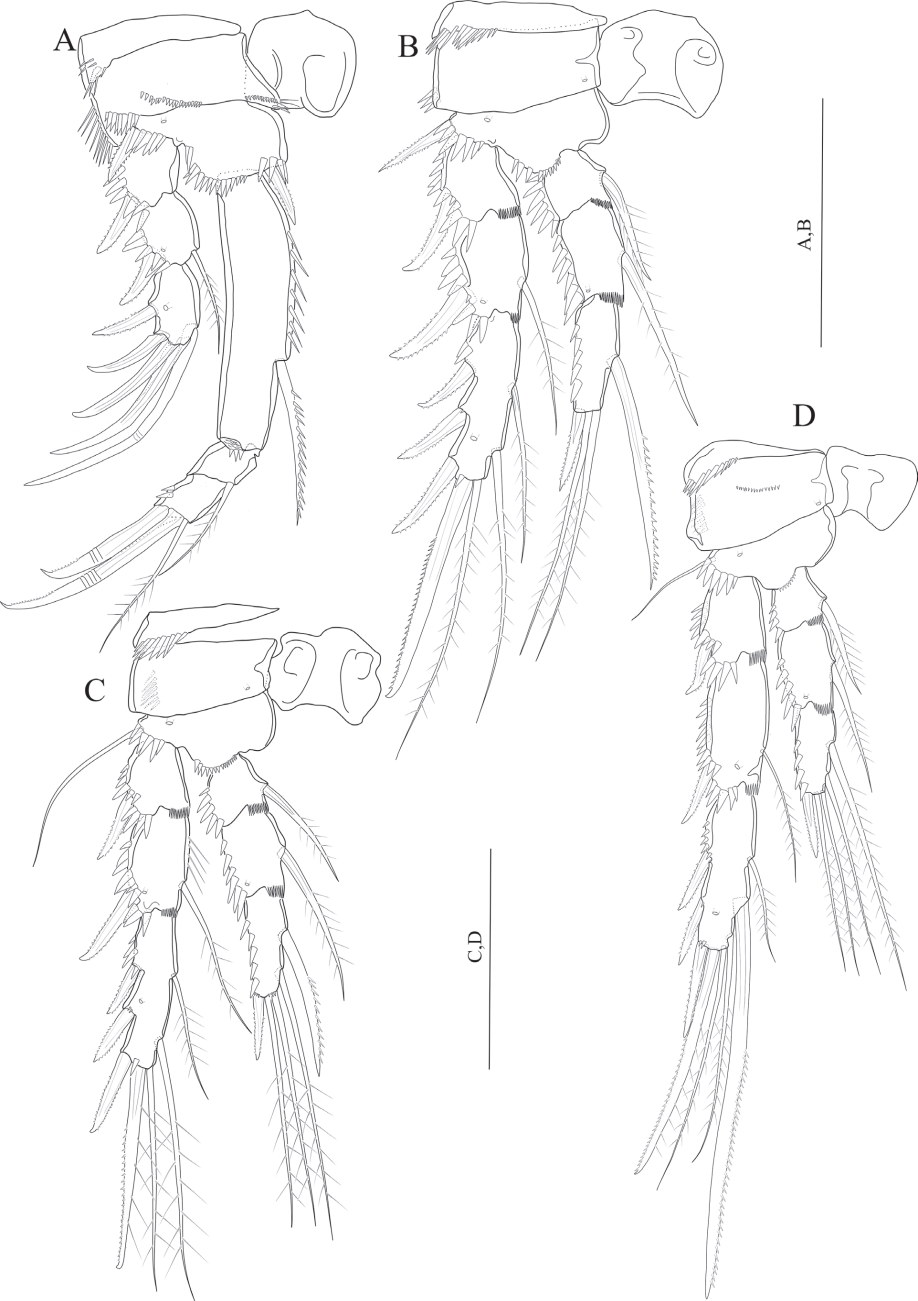
*Nitocrasonmezi* sp. nov. female holotype **A**P1, anterior **B**P2, anterior **C**P3, anterior **D**P4, anterior. Scale bars: 50 μm.

P1–P4 (Fig. 11A–D) exopod and endopod three-segmented. Intercoxal sclerite squarish and unornamented. Praecoxa triangular, outer margin with row of spinules. Exp-1 without inner seta.

P1 (Fig. 11A) same as in *N.loweae* sp. nov. except for basis without spinules along inner margin; inner margin of exp-1, 2 unornamented; innermost geniculate seta of exp-3 naked; exopod reaching slightly above the middle of enp-1 and aligned with the insertion of the inner seta of enp-1; enp-1 ~ 4.3 × as long as maximum width; subdistal seta of enp-1 unipinnate and located more proximally than that of *N.loweae* sp. nov. enp-2 without spinules on outer distal margin; enp-3 with two small spinules on outer margin.

P2 (Fig. 11B) similar to that of *N.loweae* sp. nov. except for exp-2 without setules along inner margin; inner seta of enp-3 unipinnate and stronger than in *N.loweae* sp. nov.

P3 (Fig. 11C) similar to that of *N.loweae* sp. nov. except for exp-2 with setules along inner margin; innermost seta of enp-3 unipinnate and stronger than in *N.loweae* sp. nov.

P4 (Fig. 11D) similar to that of *N.loweae* sp. nov. except for exp-2 without setules along inner margin; middle inner seta of exp-3 unipinnate, longest and stronger than that of *N.loweae* sp. nov.; innermost seta of enp-3 longest and plumose.

P5 (Fig. 9D) similar to that of *N.loweae* sp. nov. except for inner baseoendopod lobe narrower and extends halfway along the exopod, innermost seta shortest (seta I); outermost seta of exopod (seta VI) longer than in *N.loweae* sp. nov.

Armature formula of swimming legs same as in *N.loweae* sp. nov.

**Male.** Unknown.

#### Etymology.

The specific name is given in honour of Associate Prof Dr Serdar Sönmez from Adıyaman University for his contribution to copepod taxonomy in Türkiye. It is a noun in the genitive case.

### ﻿ Nitocra alperi sp. nov.

Taxon classificationAnimaliaHarpacticoidaAmeiridae

C47146B3-7080-5A85-ABAE-AE832C81F02B

https://zoobank.org/A86AFF9C-0926-4A71-8E83-040A556CF894

Figs 12 , 13 , 14

#### Type material.

***Holotype***: India • 1 ♀ (dissected on 7 slides) (NHMUK reg. no. 2023.0000). Indian Ocean, Aldabra; large tide salted lagoon; W. of Point Hadroul. Coll. Pres. K.G. Mc Kenzie, 1968; J.B.J. Wells det. (material originally registered as *N.affinis* under NHMUK reg. no. 1972.6.13.17-21).

#### Description

**Figure 12.**
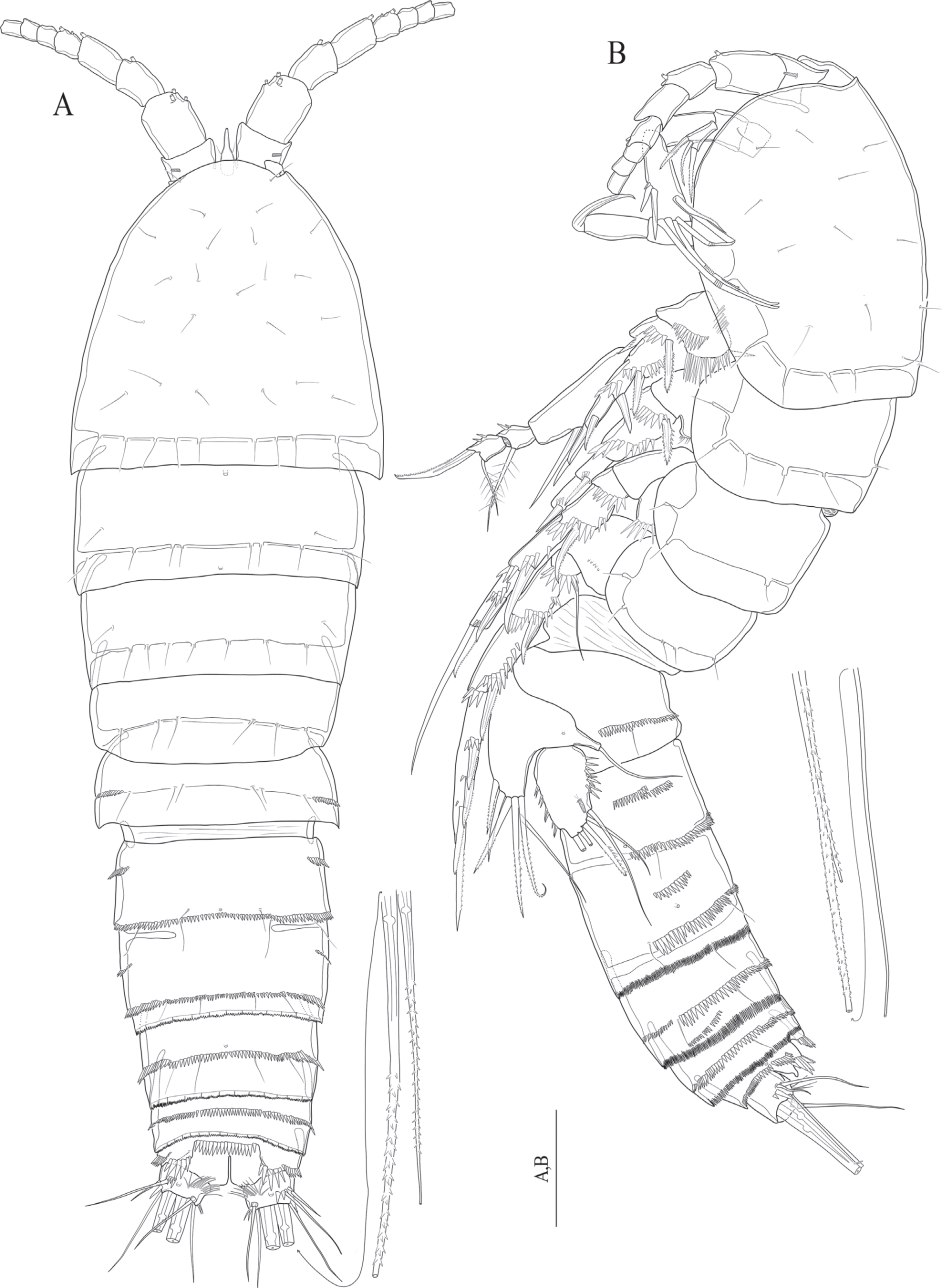
*Nitocraalperi* sp. nov. female holotype **A** habitus, dorsal **B** habitus, lateral. Scale bars: 50 μm.

**(adult female holotype).** Body (Fig. 12A, B) similar to *N.loweae* sp. nov., except for total body length 476 μm (*n* = 1) measured tip of the rostrum to posterior end of the caudal rami. Pores and sensilla as figured (Fig. 12A, B).

**Figure 13.**
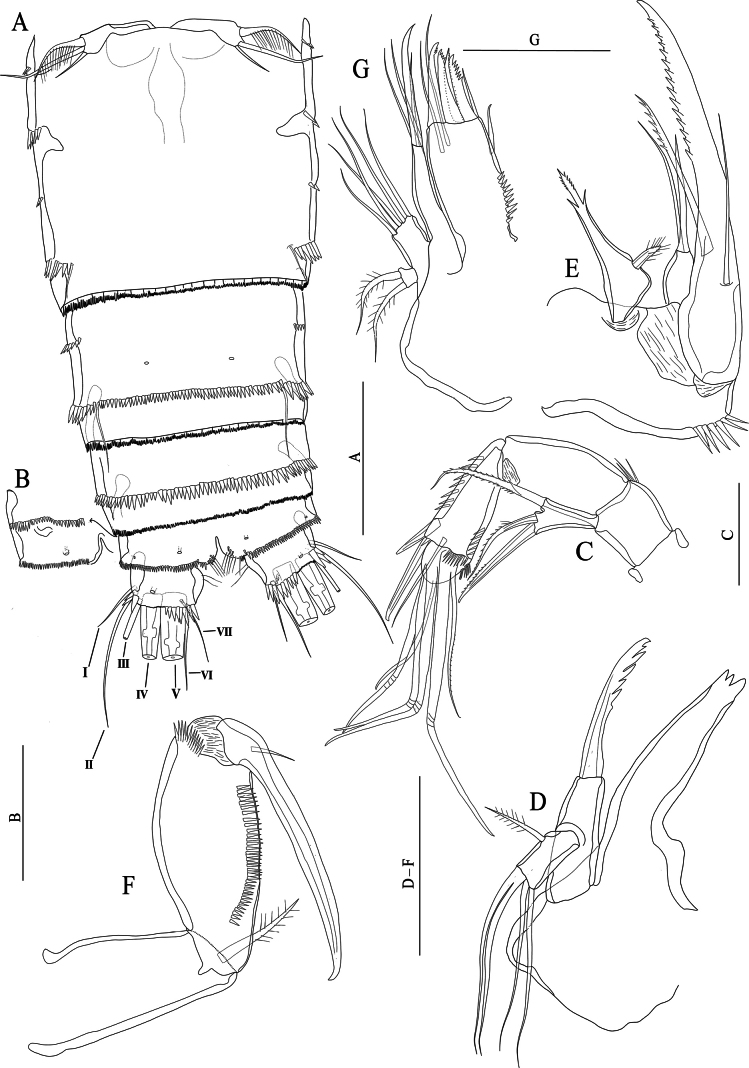
*Nitocraalperi* sp. nov. female holotype **A** urosome, ventral (P5-bearing somite omitted) **B** right part of anal somite showing spinule ornamentation, ventral **C** antenna **D** mandible **E** maxilla **F** maxilliped **G** maxillule. Scale bars: 50 μm (**A–C**); 25 μm (**D–G**).

Anal somite (Figs 12A, 13A, B), with anal operculum bearing seventeen posterior spinules; with row of robust spinules flanking each side of the anal operculum; with a posterior row of small spinules, and two pair of pores on ventrally.

Antennule eight-segmented as in *N.affinis.* Setal formula 1-[1, plumose], 2-[8 +1 plumose], 3-[8], 4-[3 +1 ae], 5-[2], 6-[3], 7-[4], 8-[5 +acrothek)]. Maximum length/maximum width ratio of antennular segments as 1:1.3:1.3:1.6:1.3:1.1:1.1:1.6.

Antenna (Fig. 13C) similar to that of *N.loweae* sp. nov. except for allobasis with spinules only on midway inner margin; subdistal seta of exopod weakly pinnate.

Mandible (Fig. 13D) similar to that of *N.loweae* sp. nov. except for exopod with four naked apical setae (two of them basally fused at base) and without spinules.

Maxilla (Fig. 13E) similar to that of *N.loweae* sp. nov. except for allobasis without spinules along convex margin near the base of endopod; endopod with one long naked seta.

Maxilliped (Fig. 13F) similar to that of *N.loweae* sp. nov. except for syncoxa unornamented.

Maxillule (Fig. 13G) similar to that of *N.loweae* sp. nov. except for coxal endite without spinule row.

**Figure 14.**
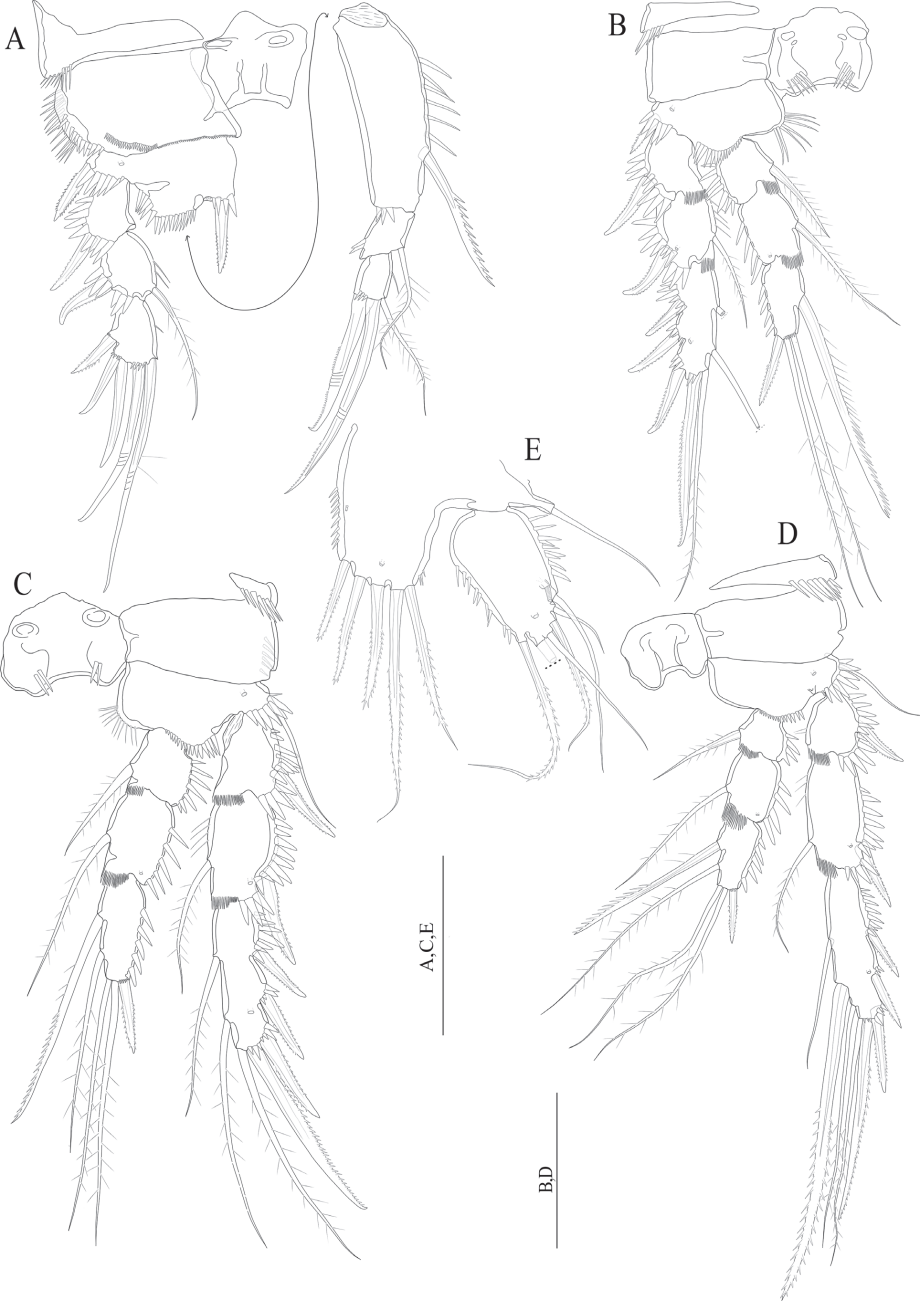
*Nitocraalperi* sp. nov. female holotype **A**P1, anterior **B**P2, anterior **C**P3, anterior **D**P4, anterior **E**P5, anterior. Scale bars: 50 μm.

P1 (Fig. 14A) similar to that of *N.loweae* sp. nov. except for coxa without spinules on/near inner margin; basis without setules along inner margin; exopod slightly extends the enp-1; enp-1 ~ 2.6 × as long as maximum width, inner margin with less spinules along inner margin, subdistal seta unipinnate and located more proximally than in *N.loweae* sp. nov.; inner margin of enp-2 with one setule, outer distal margin with few fine spinules not extending to inner margin.

P2 (Fig. 14B) similar to that of *N.loweae* sp. nov. except for intercoxal sclerite with spinules on anterior surface; basis with setules along inner margin; inner seta of enp-3 uni-plumose and stronger than in *N.loweae* sp. nov.

P3 (Fig. 14C) similar to that of *N.loweae* sp. nov. except for intercoxal sclerite with spinules on anterior surface; basis with setules along inner margin; exp-2 with setules along inner margin; innermost seta of enp-3 unipinnate and stronger than in *N.loweae* sp. nov.

P4 (Fig. 14D) similar to that of *N.loweae* sp. nov. except for exp-1 with two fine setules along inner margin; middle inner seta of exp-3 bipinnate distal half, longest and stronger than that of *N.loweae* sp. nov.; innermost seta of enp-3 uni-plumose and stronger than in *N.loweae* sp. nov.

P5 (Fig. 14E) similar to that of *N.loweae* sp. nov. except for inner baseoendopod lobe reaching middle of the exopod; exopod tapering apically, and ~ 1.5 × as long as maximum width, outermost seta much longer, seta next to outermost seta slender and naked.

Armature formula of swimming legs (not shown) same as in *N.loweae* sp. nov.

**Male.** Unknown.

#### Etymology.

The specific name is given in honours of Associate Professor Dr Alp Alper from Balıkesir University for his contribution to copepod taxonomy. It is a noun in the genitive case.

### ﻿ Nitocra serdarsaki sp. nov.

Taxon classificationAnimaliaHarpacticoidaAmeiridae

4FF95DD9-10F2-55F7-84C6-5EC14312F275

https://zoobank.org/75F4D4A1-3CE2-404E-974D-D11A1F9D4982

Figs 15 , 16 , 17

#### Type material.

***Holotype***: Türkiye • 1 ♂ (dissected on 7 slides) (reg. no. TCRC-2013/16). Ertuğrul Bay, Seddülbahir Beach; 40°2.5608'N, 26°11.0772'E; 29/09/2013; Drs Serdar Sak, Alp Alper, Orkan Metin Leg. This specimen was previously deposited in the collection of Biology Department of Balıkesir University which was labelled as *N.affinis* as a result of the faunistic project from Saros Bay, under the project number TÜBİTAK TBAG-212T105).

#### Description

**Figure 15.**
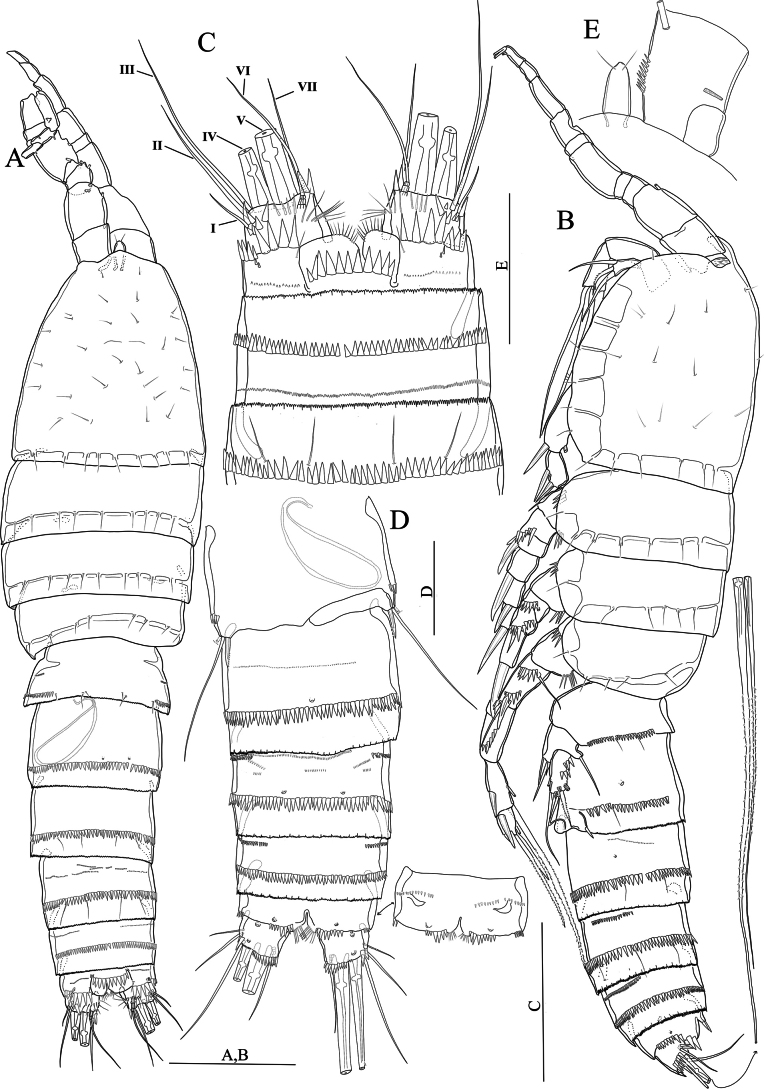
*Nitocraserdarsaki* sp. nov. male holotype **A** habitus, dorsal **B** habitus, lateral **C** penultimate and anal somites, dorsal **D** urosome, ventral (P5-bearing somite omitted) **E** rostrum and first segment of antennule. Scale bars: 50 μm.

**(adult male holotype)**: Body (Fig. 15A, B) semi-cylindrical, total body length measured from tip of the rostrum to posterior end of the caudal rami 582 μm (*n* = 1). Sensilla and pores as figured (Fig. 15A–D). Rostrum small, with two sensilla on distal margin, without rostral extension apically (Fig. 15E). Anal somite with two sensilla on both sides of anal operculum; posterior end covered with robust spinules; inner distal and lateral margin with small spinules and a pair of pores medially on ventral surface. Anal operculum (Fig. 15C) with eleven robust spinules along posterior margin. Caudal rami small and squarish; with transverse fine setules dorsally extending inner margin dorsally, and with a row of spinules laterally; ventrally with pores on near anterior and posterior margin (Fig. 15D).

**Figure 16.**
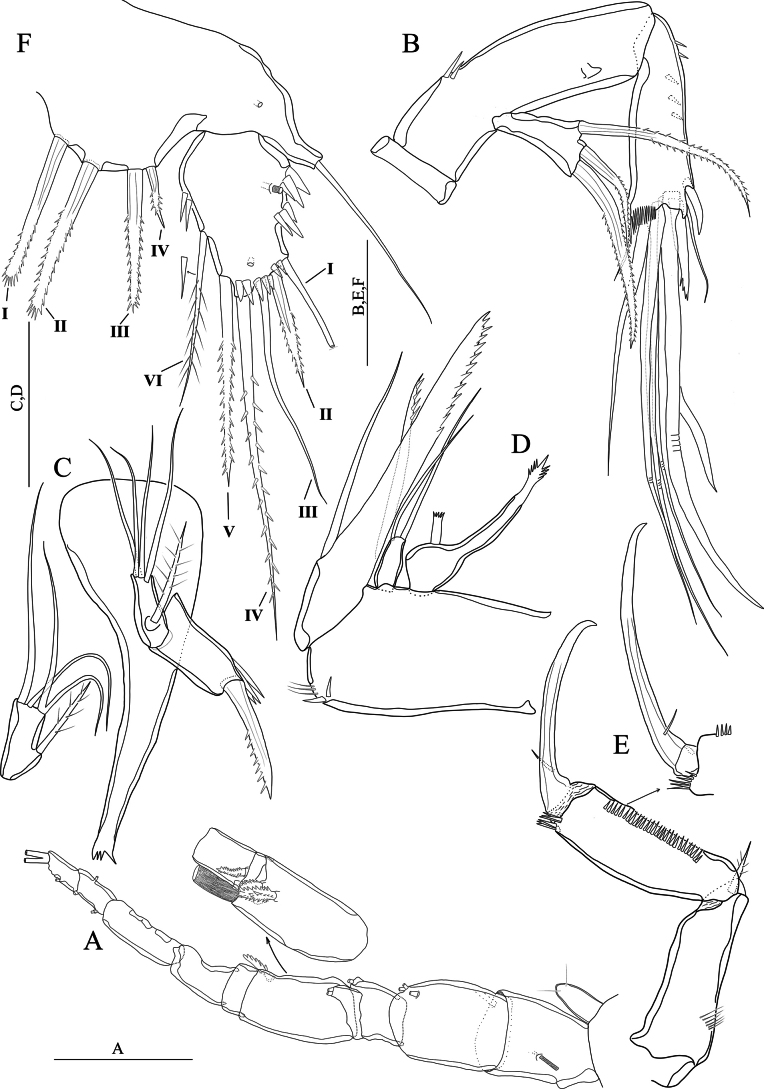
*Nitocraserdarsaki* sp. nov. male holotype **A** antennule **B** antenna **C** mandible **D** maxilla **E** maxilliped **F**P5, anterior. Scale bars: (**A**) 50 μm; (**C–F**) 25 μm.

Antennule (Fig. 16A). Setal pattern and structure similar to that of *N.loweae* sp. nov. except for segments weaker developed than in *N.loweae* sp. nov.

Antenna (Fig. 16B) comprising coxa, allobasis, one-segmented endopod and one- segmented exopod similar to that of *N.loweae* sp. nov. except for allobasis with spinules only on the middle of inner margin; spinule row along inner margin of free endopodal segment; more sparsely distributed than in *N.loweae* sp. nov.; inner apical seta of free endopodal segment 1.5 × as long as the adjacent apical seta; subdistal seta of exopod weakly pinnate.

Mandible (Fig. 16C) similar to that of *N.loweae* sp. nov. except for exopod with four naked apical setae (two of them fused basally) and without spinules.

Maxilla (Fig. 16D) similar to that of *N.loweae* sp. nov. except for allobasis without spinules along convex margin near the base of endopod; endopod with one long seta.

Maxilliped (Fig. 16E) similar to that of *N.loweae* sp. nov. except for syncoxa ~ 2.6 × as long as maximum width; basis ~ 2.7 × as long as maximum width.

Maxillule similar to that of *N.loweae* sp. nov.

**Figure 17.**
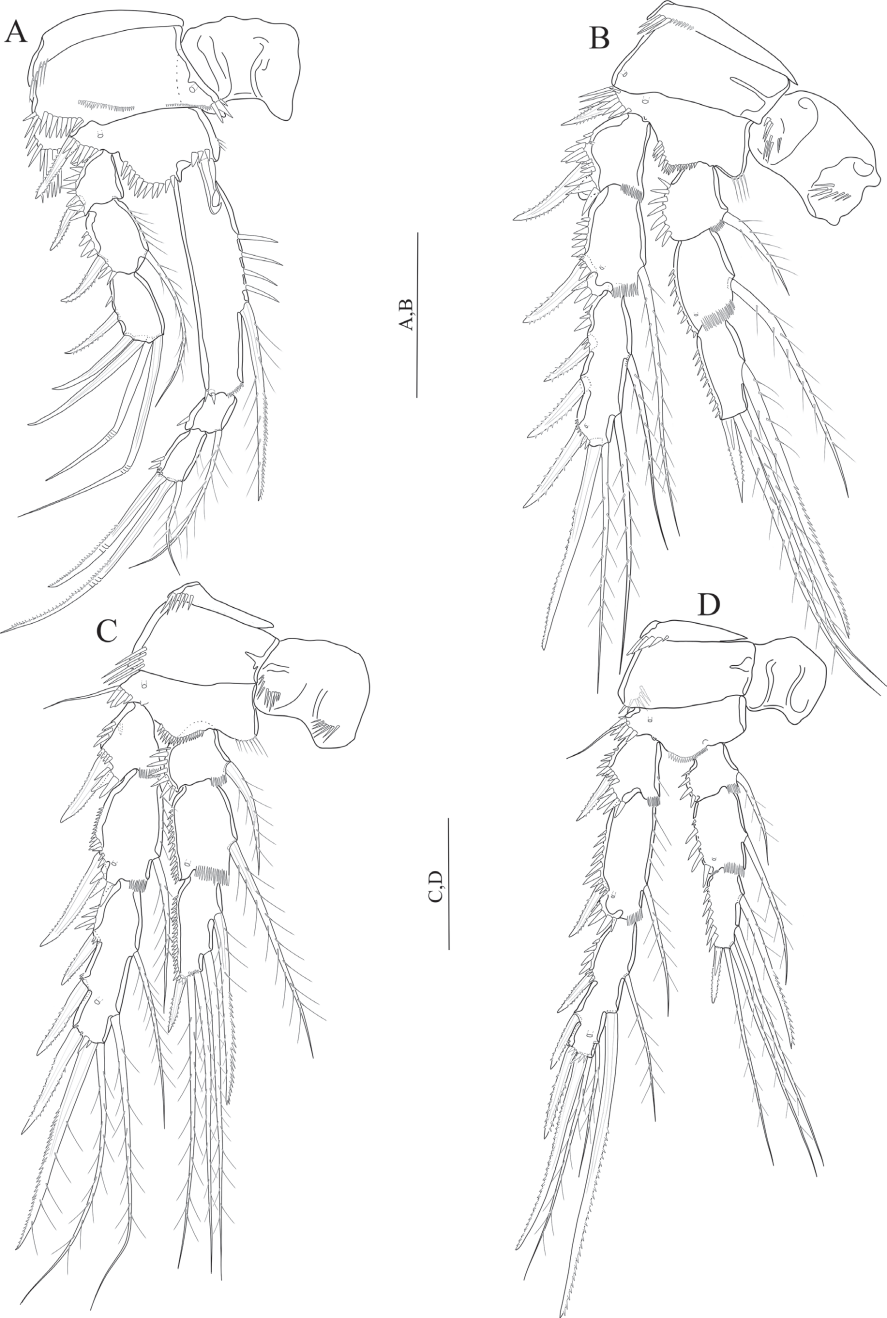
*Nitocraserdarsaki* sp. nov. male holotype **A**P1, anterior **B**P2, anterior **C**P3, anterior **D**P4, anterior. Scale bars: 50 μm.

P1 (Fig. 17A) similar to that of *N.loweae* sp. nov. except for exopod extends the level of inner seta of enp-1; enp-1 ~ 4.5 × as long as maximum width, inner margin with four well-developed spinules along inner margin, subdistal unipinnate seta located more proximally than in *N.loweae* sp. nov.; exp-3 with one spinule on outer proximal margin.

P2 (Fig. 17B) similar to that of *N.loweae* sp. nov. except for intercoxal sclerite with spinules on anterior surface; coxa unornamented; basis with setules along inner margin; inner margin of exp-3 unornamented; spinules along outer margin of exp-2, 3 weaker than in *N.loweae* sp. nov.; inner seta of enp-3 unipinnate at distal half and stronger than in *N.loweae* sp. nov.

P3 (Fig. 17C) similar to that of *N.loweae* sp. nov. except for intercoxal sclerite with spinules on anterior surface; basis with setules along inner margin; Innermost seta of enp-3 unipinnate at distal half and stronger than in *N.loweae* sp. nov.; inner apical seta naked and as long as outer spine.

P4 (Fig. 17D) similar to that of *N.loweae* sp. nov. except for spinulose row near the base of endopod weakly developed; exp-1 with two fine setules along inner margin; middle inner seta of exp-3, longest and stronger than that of *N.loweae* sp. nov.; innermost seta of enp-3 stronger than in *N.loweae* sp. nov.; subdistal inner seta of enp-3 shorter than in *N.loweae* sp. nov.

P5 (Fig. 16F) similar to that of *N.loweae* sp. nov. except for baseoendopod with four bipinnate setae, the outermost seta short and ~ 1/2 as long as the other setae.

**Female.** Unknown

#### Etymology.

The specific name is given in honour of Prof Dr Serdar Sak from Balıkesir University for his contribution to copepod taxonomy in Türkiye. It is a noun in the genitive case.

## ﻿Discussion

The family Ameiridae ranks third within the order Harpacticoida in terms of species number (Boxshall and Halsey 2004). Members of the family inhabit a wide range of sediment types and occur in virtually all salinity regimes besides living as associates of some invertebrates (Karanovic et al. 2015). The taxonomy and phylogeny of the family Ameiridae are still problematic due to numerous ill-defined genera and lack of detailed descriptions of a great deal of species (Conroy-Dalton and Huys 1996, 1998; Karanovic et al. 2015). The genus *Nitocra*, which also has a notoriously challenging and problematic taxonomy, is the largest ameirid genus, at present comprising 81 valid species and subspecies (Karanovic et al. 2015; Huys 2021; Fuentes-Reinés et al. 2022). Several factors are responsible for the origin of the current taxonomic confusion surrounding the genus *Nitocra*. The primary issue lies in the classification of *Nitocra*, which encountered initial difficulties when Boeck (1865) provided an insufficient definition of the genus, offering only fragmentary descriptions devoid of illustrations. Furthermore, the majority of conventional marine genera described and outlined in the early years of the 20^th^ century (Sars 1907, 1911a, b, c; Lang 1935, 1936) did not substantially enhance the precise delineation of either the type genus *Ameira* or the genus *Nitocra*. The third factor blurring the generic boundary of the genus *Nitocra* is the arbitrary addition of new species, which resulted in the amalgam of phylogenetically unrelated species into the genus. Due to the taxonomic confusion within the genus *Nitocra* arising from these factors, resolving the complexity through a single study has become an impossible task. Consequently, urgently conducting modern standard redescriptions of numerous species within the genus with insufficient descriptions will significantly contribute to solving the problem (Karanovic and Pesce 2002). Therefore, within this scope, the redefinition of *N.affinis*, one of the important polytypic species in the genus, based on lectotype material in this study has provided significant contributions to resolving the *N.affinis* species complex and solving the taxonomic problem within the genus. On the other hand, it is crucial to delineate species groups within the genus from a phylogenetic perspective. Despite being the largest genus within the family Ameiridae, with a notoriously difficult and problematic taxonomy, no attempts have been made to delineate species groups within the genus *Nitocra* until Gómez et al. (2012) who attempted to establish species groups within the genus *Nitocra* in order to ground the classification of the genus on a phylogenetic basis although they have not conducted a phylogenetic tree-based study. Gómez et al. (2012) recognised three species groups based on the setal formula of P1 exp-2, 3. The first group of species contained *Nitocrasewelli* and *N.platypusbakeri* which bear one inner seta on P1 exp-2 and four setae on P1 exp-3; the second group of species comprised *N.reductareducta*, *N.delaruei*, *N.blochi*, *N.gracilimana*, *N.phlegrea*, and *N.chelifer* which lack an inner seta on P1 exp-2, but bear five setae on P1 exp-3; the third group of species accommodated the rest of the species with one inner seta on P1 exp-2 and five setae on P1 exp-3. Gómez et al. (2012) also recognised a distinct subgroup of species within the third group, based on the setal formula of P2–P4 exp-3 (7-7-7), P2–P4enp-3 (4-5-5), and P2–P4enp-1 (1-1-1). *Nitocraaffinis*, *N.colombiensis*, *N.rijekana*, *N.stygia*, *N.hamata*, *N.elegans*, *N.sonmezi* sp. nov., *N.loweae* sp. nov., *N.serdarsaki* sp. nov., and *N.alperi* sp. nov., constitute another subgroup of species within the group which can be called the *affinis* group on the basis of a) by having a spinulose, long, spine-like inner middle seta on P4 exp-3, b) by having the same setal formula on the P2–P4 exp-3 (7-7-8) and P2–P4enp-3 (4-5-5), c) by the number of seta on the P2–P4 exp-2, P2–P4enp-1, 2 (1-1-1), d) by the elongated P1enp-1.

*Nitocraaffinis* is clearly distinguished from other congeners in the *affinis* group by the combination of the following characters in female; rostral projection reaching ~ 1/2 of the rostral length, anal operculum with 14 spinules, the reduced maxillary endopod with one slender seta, inner middle seta of P4 exp-3 strongly spinulose and long, female P5 baseoendopod with five and exopod with six setae respectively. Male P5 baseoendopod with four and exopod with six setae, respectively. *Nitocraaffinis* has subsequently been reported from several other localities: Willey (1930) recorded it from Mangrove Lake in Bermuda, presenting only the setal formula of swimming legs and the number of spinules (fifteen) on the anal operculum. Vervoort (1964) reported *N.affinis* from Ifaluk Atoll in the Pacific Ocean, describing the P4 exp-3 middle inner seta as strong and long, anal operculum as spinulose, antenna exopod as one-segmented, and P1enp-1 as slightly longer than the exopod. Unfortunately, the specific identity of the above-mentioned populations cannot be verified due to insufficient morphological data and should therefore be considered as unverified records. However, considering the isolated environments of *Nitocraaffinis* from Bermuda and Ifaluk Atoll, it is strongly possible that each of these populations of *N.affinis* may represent distinct species.

Rajthilak et al. (2015) recently recorded *Nitocraaffinis* from South-east India, providing both morphological and molecular data. But, based on the morphological information provided by Rajthilak et al. (2015), even the Ameiridael identification of the south-east Indian population of *N.affinis* is uncertain and therefore this record cannot be verified.

It has been demonstrated several times that many so-called cosmopolitan harpacticoid species in fact represent species complexes (Gómez et al. 2012; Karanovic et al. 2015; George 2018; Karaytuğ et al. 2021). Vervoort (1964) and Fuentes-Reinés and Suárez‐Morales (2014) proposed the potential existence of a species complex within *N.affinis*. Through comprehensive morphological assessments of specimens gathered from diverse geographical locations initially labelled as *N.affinis*, this investigation has unveiled the presence of four new species in this study, thus affirming *N.affinis* as a species complex.

Lack of original descriptions or insufficient taxonomic information are the main reasons for the formation of species complexes. The swimming legs segmentation and their setal formula, the number of segments in the antennule and the structure of antennary exopod are the most commonly used morphological characters for delineation of the species in harpacticoid taxonomy (Wells 2007). Therefore, in recent studies, most researchers have concentrated on finding microcharacters such as spinule ornamentation (Alper et al. 2023) or pore signature (Karanovic et al. 2015; Karanovic and Cho 2016) which proved to be helpful in differentiating closely related morphospecies. So, it was not surprising that similar results emerged in the ameirid taxonomy. For example, Karanovic and Cho (2012) distinguished two *Ameira* species based on setules, spinules or pore ornamentations on the somites or appendages. In this study, within the *N.affinis* complex four new species namely *N.sonmezi* sp. nov., *N.serdarsaki* sp. nov., *N.alperi* sp. nov., and *N.loweae* sp. nov., were revealed mostly based on micro-morphological characters which, once again, demonstrated their importance in copepod taxonomy.

*Nitocraloweae* sp. nov. was collected from Brighton and identified as *N.affinis* (Ventham 2011). *Nitocraloweae* sp. nov. is described on the basis on one female and a male specimen and can be easily distinguished from other congeners in the *affinis* group by (a) the robust, spinulose ornamentations of urosomites, (b) by the presence of twelve large dorsal spinules on the anal operculum, c) the maxilla endopod with two slender setae. Details of the specific differences are given in Table 1. *Nitocraloweae* sp. nov. and *N.affinis* can be easily differentiated by the following characters: the antennule of *N.loweae* sp. nov. differs from that of *N.affinis* in having two plumose setae and one plumose seta on the second and third segment, respectively; the two inner setae on the seventh segment set close to each other in *N.affinis* but widely separated in *N.loweae* sp. nov.; the proximal-most inner seta on the eighth segment in *N.affinis* is located more distally than that of *N.loweae* sp. nov.; *N.loweae* sp. nov. lacks the rostral projection on the rostrum and has five setae on the P5 baseoendopod of the male, whilst according to Gurney’s (1927) description, *N.affinis* has four setae on the P5 baseoendopod of the male. *Nitocraloweae* sp. nov. has four naked distal setae and two naked lateral setae on the mandibular endopod, and two slender setae on the maxilla endopod, whereas all other species in the *affinis* group have four naked distal setae and one lateral seta on the mandibular endopod, and one slender seta on the maxilla endopod. In comparison to other species within the *affinis* group, *N.loweae* sp. nov. is regarded as exhibiting a more primitive state with respect to these characters.

**Table 1.** Differentiating characters of the affinis species group. +: present; -: absent; ?: unknown.

	number of spinules on anal operculum	apical rostral projection	♂ P5 setae on endopod and exopod of male	inner ornamentation of the caudal rami	ornamentation of the inner seta of P1enp-1	Body length (mm) ♀	Body length (mm) ♂	seta of mandibular endopod	seta of maxillary endopod
***N.affinis***	14	+	4:6	fine setules	bipinnate	0.61 (Gurney 1927)	0.48 (Gurney 1927)	4 naked on distal; 1 plumose on lateral	1 long seta
***N.californica* Lang, 1965**	14	-	4:6	fine setules	plumose	0.70	?	4 naked on distal; 1 plumose on lateral	1 long seta
***N.colombiensis* Fuentes-Reinés, Suárez-Morales, 2014**	16	+	3:6	small, spinules	plumose	0.70	0.51	4 naked on distal; 1 plumose on lateral	1 long seta
***N.stygia* Por, 1968**	20	?	4:6	?	plumose	0.40	?	?	?
***N.rijekana* Petkovski, 1954**	18	?	5:6	?	?	0.60	0.50	?	?
***N.sonmezi* sp. nov.**	15	-	?	fine setules	unipinnate, spine-like	0.40	?	4 naked on distal; 1 naked on lateral	1 long seta
***N.serdarsaki* sp. nov.**	11	-	4:6	fine setules	semiplumose-semipinnate	?	0.58	4 naked on distal; 1 plumose lateral	1 long seta
***N.alperi* sp. nov.**	17	+	?	fine setules	unipinnate, spine-like	0.47	?	4 naked on distal; 1 plumose on lateral	1 long seta
***N.loweae* sp. nov.**	12	-	5:6	robust spinulose	plumose	0.57	0.38	4 naked on distal; 2 naked on lateral	2 long setae

*Nitocrasonmezi* sp. nov. was described on the basis on one female specimen from mediolittoral zone of coast of Hatay, Turkey. *Nitocrasonmezi* sp. nov. is differentiated from other species of the *affinis* group by (a) the number of spinules on the anal operculum, (b) in the shape of P1enp-1 which is ~ 4.3 × as long as maximum width, (c) by the ornamentation of the subdistal inner seta of P1enp-1, (d) in the ornamentation of P5 endopodal and exopodal setae, e) in the ornamentation of urosomites and (f) in the ornamentation of the setae of P2–P4. Details of the specific differences are given in Table 1.

*Nitocraalperi* sp. nov. was identified as *N.affinis* from the Indian Ocean (Wells and Rao 1987). *Nitocraalperi* sp. nov. can be distinguished from other congeners of the *affinis* group by (a) the total length of P4 endopod segments; (b) the ornamentation of the inner seta P2–P4enp-3; (c) the ornamentation of the inner seta of P1enp-1, length to width ratio of this segment; (d) the surface ornamentation of somites and number of spinules on the anal operculum (seventeen) (see Table 1 for detailed comparisons). *Nitocraalperi* sp. nov. shares the rostral projection on its rostrum with *N.affinis*, *N.colombiensis*, and *N.loweae* sp. nov.

*Nitocraserdarsaki* sp. nov. was identified from the Aegean coast of Türkiye on the basis of the one male specimen. *Nitocraserdarsaki* sp. nov. can be distinguished from other new species by (a) the number of spinules on the anal operculum; (b) the ornamentation of P5 exopod and baseoendopod; (c) the ornamentation of inner setae of P2–P4 endopod-3, (d) the P1enp-1 inner seta ornamentation; (e) the length and (f) ornamentation of the middle inner seta of P4 exp-3 (see Table 1 for detailed comparisons). *Nitocraaffinis* has four setae on the P5 baseoendopod of the male, whereas *N.serdarsaki* sp. nov. has five setae on the P5 baseoendopod of the male.

These new species can also be easily distinguished from *N.hamata* and *N.elegans* which are in the *affinis* group, by following characters; (a) shape of female P5 exopod, (b) ornamentations of the abdominal somites and (c) the ornamentations of mouthparts (Bodin 1970; Gee 2009). *Nitocrahamata* is distinguished from other *affinis* species group by the shape of female P5 exopod which is longer and slender (Bodin 1970), P1enp-1 length and the structure of P3enp-3.

The status of subspecific taxa within the genus *Nitocra* (*N.reductafluviatilis* and *N.sewellihusmanni*) have been revised by Gómez et al (2012) to recognise them as species, based on consistent morphological differences. In this context, we have also re-evaluated the status of the subspecies of *N.affinis* below:

### ﻿Establishment of *Nitocrarijekana* Petkovski, 1954

*Nitocrarijekana* was originally described as a form of *N.affinis* by Petkovski (1954) from Rijeka, Northern Adriatic (Mediterranean Sea), and has not been recorded since its original description. In the same study, Petkovski (1954) examined the material of *N.affinis* from Dubrovnik and compared it with that of Rijeka, determining significant morphological differences between them. The differences between *Nitocrarijekana* and *N.affinis* are as follows: (a) inner middle seta of P4 exp-3 of *Nitocrarijekana* is not as long as and not as strong as that of *Nitocraaffinis*, (b) the male P5 baseoendopod with five and exopod with six setae (in *Nitocraaffinis* 4:6). *Nitocrarijekana* can also be easily distinguished from its congeners by having a long but plumose inner middle seta of P4 exp-3. We believe that morphological differences between *Nitocraaffinisrijekana* Petkovski, 1954 and *Nitocraaffinis* are significant enough to warrant upgrading *Nitocraaffinisrijekana* to a specific rank. The detailed comparison is provided in Table 1.

### ﻿Establishment of *Nitocracalifornica* Lang, 1965

Lang (1965) originally described *Nitocraaffiniscalifornica* in a tidal pool from Monterey Bay, California. Later on, Kunz (1975) recorded *Nitocraaffiniscalifornica* from Gonubie, South Africa, by examining 62 specimens collected from the reef area. Kunz (1975) observed variabilities on the P5, which may indicate that Kunz (1975) was dealing with more than one species. Unfortunately, Kunz (1975) only described the P1 and P5, thus making it impossible to confirm the specific status of Kunz’ (1975) specimens. After Kunz (1975)Apostolov (1980) recorded *N.affiniscalifornica* from Bulgaria. Fuentes-Reinés and Suárez-Morales (2014) mentioned that the Bulgarian and South African specimens may represent different species. This observation is supported by the notably shorter P1 exopod found in both the Bulgarian and South African specimens, whose exopodal ramus extends to ~ ¾ of the length of the first endopodal segment, distinctly deviating from the characteristic of *N.a.californica*, where the exopod and the first endopod segment exhibit equal lengths.

Lang’s (1965) subspecies is here upgraded to full species rank since it differs sufficiently from Gurney’s (1927) population and its congeners to warrant such status on the basis of the following characters: (a) P1 exp-3 ~ as long as exopod and exceeds the origin of P1enp-1 inner seta (in all other species, the P1 exp-3 does not exceeds the origin of P1enp-1 inner seta); (b) the setal ornamentation of swimming legs; (c) male P5 baseoendopod with four setae. Detailed comparison is provided in Table 1.

### ﻿Establishment of *Nitocrastygia* Por, 1968

Por (1968) described *Nitocraaffinisstygia* from land-locked basins in the Red Sea. In the original description, the female P5, the abdominal segment, the caudal rami, P1, P4 exopod and male P5 were illustrated. Por’s (1968) subspecies is here upgraded to full species rank since it differs sufficiently from Gurney’s (1927) population and its congeners to warrant such status on the basis of the following characters: (a) large hyaline field on female P5 and its large dimensions, (b) inner middle seta of P4 exp-3 is not as long as and not as strong as in *N.affinis*, (c) penultimate somite surrounded by spinule (only from dorsal to lateral in *N.affinis*), (d) female baseoendopodal setae are almost equal in length, (e) male baseoendopod with four setae. Detailed comparison is provided in Table 1.

### ﻿Establishment of *Nitocracolombiensis* Fuentes-Reinés & Suárez‐Morales, 2014

*Nitocracolombiensis* is originally described as *Nitocraaffiniscolombiensis* from a lagoon in Colombia (Fuentes-Reinés and Suárez‐Morales 2014). This subspecies from Colombia is here upgraded to full species rank since it differs sufficiently from Gurney’s (1927) population and its congeners to warrant such status on the basis of the following characters: (a) number of setae of the male P5 endopod of; (b) number of spinules on the anal operculum; (c) shape and ornamentation of the middle inner seta P4 exp-3; (d) ratio of the P1enp-1; (e) body ornamentation; (f) antennular setal formula; (g) maxillule basis with four setae. In the original description of *Nitocracolombiensis*, apical rostral projection is given as diagnostic character for the species. But the rostral projection is also observed both in *N.affinis* and *N.alperi* sp. nov.

Fuentes-Reinés and Suárez-Morales (2014) provided an identification key for *affinis* group. The key distinguished *Nitocracolombiensis* from other species with the apical rostral projection. We observed the apical rostral projection both in *N.affinis* and *N.alperi* sp. nov. in this study. Therefore, here we revised the identification key for taxa contained in the *N.affinis* group.

### ﻿A key to the *Nitocraaffinis* species group

**Table:** 

1	Inner middle seta of P4 exopod-3 not strong and longer than other setae	***N.rijekana***
–	The inner middle seta of P4 exopod-3 long, strong and spinulose	**2**
2	Rostrum with rostral projection	**3**
–	Rostrum without rostral projection	**4**
3	P1enp-1 with a plumose inner seta; male P5 baseoendopod with 3 setae	***N.colombiensis***
–	P1enp-1 with a spinulose inner seta; male P5 baseoendopod with 4 setae	***N.affinis***
–	P1enp-1 with unipinnate seta	***N.alperi* sp. nov.**
4	P1 exopod not reaching beyond the insertion site of the inner seta of P1enp-1; male P5 baseoendopod with 4 setae	***N* . *californica***
–	P1 exopod reaching beyond the insertion site of the inner seta of P1enp-1	**5**
–	P5 baseoendopod with large hyaline field; male P5 baseoendopod with 4 setae	***N.stygia***
–	P5 baseoendopod without large hyaline field	**6**
5	Mandibular endopod with 1 plumose seta laterally, and 5 naked setae apically	**7**
–	Mandibular endopod with 1 plumose seta laterally, and 4 naked setae apically	***N.serdarsaki* . sp. nov.**
6	Penultimate somite with robust spinules on ventral, spinules on lateral side (not surrounded dorsoventrally); caudal rami inner margin covered with robust spinules; inner proximal seta P3 endopod-3 naked and not longer than other	***N.loweae* sp. nov.**
7	Penultimate somite ornamented with spinules along somite (surrounded dorsoventrally) ventral; caudal rami inner margin naked; inner proximal seta of P3 endopod-3 not longer than other setae and unipennate	***N.sonmezi* sp. nov.**

## ﻿Conclusion

The growing significance of microcharacters in copepod taxonomy has revealed that numerous species lacking comprehensive descriptions are, in fact, part of species complexes. In this study, *Nitocraaffinis* was redescribed based on lectotype material which facilitated us through detailed comparison with specimens recorded and labelled as *N.affinis* from distantly related localities. The results clearly indicated that each of these specimens attributed to *N.affinis* corresponds to a distinct species. Four new species have been described from different localities, and named as *N.sonmezi* sp. nov., *N.loweae* sp. nov., *N.alperi* sp. nov., and *N.serdarsaki* sp. nov. The status of subspecific taxa of *N.affinis* has been re-evaluated based on the literature and four subspecies of *N.affinis* have been reinstated to specific rank, and named as *N.stygia*, *N.rijekana*, *N.californica*, and *N.colombiensis*. The description of the majority of the species/subspecies within the *Nitocra* genus is notably insufficient. While the morphological examination of mouthparts in ameirid taxa can be challenging, a detailed morphological analysis of mouthparts may significantly contribute to resolving the problematic taxonomy of the genus. Indeed, in this study, although setal formulae of the swimming legs of the four newly described species are the same as in *N.affinis* species, new morphological differences have been detected. For instance, there is a distinct apical extension of the rostrum of *N.affinis* and *N.alperi*. While in *N.loweae*, the maxilla endopod is represented by two setae of equal length, it is represented by a single seta in other species within the *affinis* group. These findings clearly underscore the significant contributions that detailed species descriptions will make to resolve the challenging taxonomy of the genus *Nitocra*. In addition to morphological studies, the phylogenetic analysis of molecular data to be obtained will provide valuable insights into both the taxonomy of the genus *Nitocra* and the phylogenetic relationships among genera within the family Ameiridae.

## Supplementary Material

XML Treatment for Ameiridae

XML Treatment for Nitocra

XML Treatment for Nitocra affinis

XML Treatment for Nitocra loweae

XML Treatment for Nitocra sonmezi

XML Treatment for Nitocra alperi

XML Treatment for Nitocra serdarsaki

## ﻿Additional information

### Author ORCIDs

Nuran Özlem Yıldız https://orcid.org/0000-0001-7129-7276

Süphan Karaytuğ https://orcid.org/0000-0001-8980-4133

### Data availability

All of the data that support the findings of this study are available in the main text.
